# Discrimination of Deletion and Duplication Subtypes of the Deleted in Azoospermia Gene Family in the Context of Frequent Interloci Gene Conversion

**DOI:** 10.1371/journal.pone.0163936

**Published:** 2016-10-10

**Authors:** Tibor Vaszkó, János Papp, Csilla Krausz, Elena Casamonti, Lajos Géczi, Edith Olah

**Affiliations:** 1 Department of Molecular Genetics, National Institute of Oncology, Budapest, Hungary; 2 Department of Chemotherapy, National Institute of Oncology, Budapest, Hungary; 3 Department of Experimental and Clinical Biomedical Sciences, University of Florence, Florence, Italy; 4 Andrology Service, Fundacio´ Puigvert, Instituto de Investigaciones Biome´dicas Sant Pau (IIB-Sant Pau), Universitat Autonoma de Barcelona, Barcelona, Spain; University of Naples Federico II, ITALY

## Abstract

Due to its palindromic setup, AZFc (Azoospermia Factor c) region of chromosome Y is one of the most unstable regions of the human genome. It contains eight gene families expressed mainly in the testes. Several types of rearrangement resulting in changes in the cumulative copy number of the gene families were reported to be associated with diseases such as male infertility and testicular germ cell tumors. The best studied AZFc rearrangement is gr/gr deletion. Its carriers show widespread phenotypic variation from azoospermia to normospermia. This phenomenon was initially attributed to different gr/gr subtypes that would eliminate distinct members of the affected gene families. However, studies conducted to confirm this hypothesis have brought controversial results, perhaps, in part, due to the shortcomings of the utilized subtyping methodology. This proof-of-concept paper is meant to introduce here a novel method aimed at subtyping AZFc rearrangements. It is able to differentiate the partial deletion and partial duplication subtypes of the Deleted in Azoospermia (DAZ) gene family. The keystone of the method is the determination of the copy number of the gene family member-specific variant(s) in a series of sequence family variant (SFV) positions. Most importantly, we present a novel approach for the correct interpretation of the variant copy number data to determine the copy number of the individual DAZ family members in the context of frequent interloci gene conversion.Besides DAZ1/DAZ2 and DAZ3/DAZ4 deletions, not yet described rearrangements such as DAZ2/DAZ4 deletion and three duplication subtypes were also found by the utilization of the novel approach. A striking feature is the extremely high concordance among the individual data pointing to a certain type of rearrangement. In addition to being able to identify DAZ deletion subtypes more reliably than the methods used previously, this approach is the first that can discriminate DAZ duplication subtypes as well.

## Introduction

Chromosome Y has a unique structure, with about 30% of the male-specific region (MSY) covered by large palindromes consisting of very long, nearly identical direct and inverted repeats called amplicons [1]. Such a structure makes the Y chromosome prone to rearrangement, especially in the Azoospermia Factor c (AZFc) domain [2]. Indeed, AZFc is one of the most unstable regions of the human genome, and numerous structures deducible from the reference sequence through inversion, deletion and duplication, or combinations thereof, have been reported [3–5].

Eight gene families, including five with protein-coding active copies, are located exclusively within the 3.5 Mb AZFc region [2]. Both decrease and increase in the gene dosage (i.e. the cumulative copy number) of gene families might be associated with diseases. The lack of the entire AZFc region has long been shown to cause azoospermia or oligozoospermia [6]. Many studies have been conducted so far to screen for partial AZFc rearrangements in relationship with male infertility with findings that are not easy to reconcile. [3,5,7–18].

According to a comprehensive review, DAZ (GeneBank accession number: NG_004755.2) and CDY1 families represent key AZFc spermatogenic determinants [19]. A meta-analysis including 18 studies with more than 12,000 men showed that gr/gr deletion occurred more frequently in infertile than control men. Furthermore, the association was dependent on ethnicity and geographic region [20]. A population-based survey of 20,000 Y-chromosomes also states that gr/gr deletion doubles the risk of severe spermatogenic failure [21].

Carriers of gr/gr deletion show extensive phenotypic variation ranging from normal spermatogenesis to azoospermia [6]. This variation was supposed to rely, at least partly, on the different AZFc deletion subtypes based on the missing *DAZ* or *CDY1* family members. Some studies supported [3,22], others refuted this hypothesis [14,15,17,23]. As far as partial AZFc duplications are concerned, one study linked them to spermatogenic impairment [12], while two others were unable to detect a significant effect of excess AZFc gene dosage on spermatogenesis [15,24]. Subtyping has never been attempted in duplication samples due to the lack of an appropriate method. Apart from spermatogenic impairment, gr/gr deletion also seems to be a risk factor for testicular germ cell tumors (TGCTs) according to a study carried out by the International Testicular Cancer Linkage Consortium (ITCLC) [25]. No subtyping studies have been conducted at all in TGCTs so far. In summary, the above findings reflect some inconsistency concerning the role of certain AZFc rearrangements in conferring susceptibility to diseases, and it is likely that selection and methodological biases in some studies are also responsible for the controversies [15,19].

**Fig 1:**
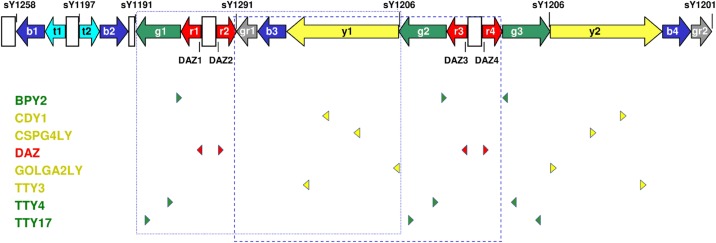
Structure of the AZFc region with the eight gene families. The colored arrows show the direct and inverted repeats of AZFc. The colored arrowheads indicate the members of the eight gene families located in the region. The two rectangles enclose the gene family members eliminated by gr/gr deletion with two different breakpoints (g1/g2 versus r2/r4 deletion), respectively. The STS markers that have usually been analyzed in search for deletions are also shown.

Partial AZFc deletions and duplications always change the cumulative copy number of the affected gene families. For example, the relatively well-characterized gr/gr deletion reduces, among others, the dose of the Deleted in Azoospermia (DAZ), the CDY1 and the BPY2 gene families by eliminating, in all cases, two members of the first and one member of the second and third family, respectively. However, the combination of the deleted gene family members may differ depending on the location of the breakpoints (Fig 1). Inversions prior to deletion may further increase the number of potential outcomes. The situation is similarly complicated in the case of duplications. Unlike other AZFc gene families, the four DAZ genes code for proteins with notably different composition [26]. Therefore, rearrangements affecting different members may confer susceptibility of different extent to male infertility and/or TGCTs. The ability to make distinction between the various combinations of deleted or duplicated gene family members, i.e. AZFc deletion and duplication subtypes, has been expected to contribute to the definition of the role of AZFc rearrangements in diseases. However, subtyping efforts, conducted so far exclusively in deletion samples to identify factors responsible for male infertility, have led to inconsistent results. It may mean that the deletion subtypes, at least those distinguished so far, do not really have a role in male infertility. But the controversy may also be the consequence of the shortcomings of the applied methods. Probably, further subtyping studies, partly in duplication samples, will be needed to reassuringly clarify the role of AZFc rearrangements in diseases. Those studies will preferably require methods that are equally efficient in both deletion and duplication subjects and, at the same time, have higher resolution then the presently used ones. We present here a new approach, called variant ratio analysis, which can be an alternative to the often-applied combination of dosage PCR and restriction fragment length polymorphism (RFLP) in AZFc subtyping.

A method suitable for subtyping in AZFc must be capable of differentiating the members of a gene family from one another; therefore, variant ratio analysis has been built upon family member-specific sequence family variants (SFVs). The ratio of the family member-specific variant to its non-specific counterpart is determined at every studied SFV position. This quantification relies on measuring the area-under-the-curve (AUC) of signals given by the specific and the non-specific variants in sequencing electropherograms. Samples are divided into partial deletion, non-rearranged (control) and partial duplication groups based on the within-sample distribution of the variant ratios. Taking the rearrangement status into account, the copy number of the specific variant can be derived from the variant ratio at each SFV position in every sample. The applicability of the family member-specific SFVs as markers, i.e. the copy numbers that unambiguously indicate the copy number of the corresponding DAZ family member, is assessed by using the control panel. This is necessary since in addition to deletion, duplication and inversion, the members of gene families located in AZFc are also subjected to interlocus gene conversion [14,27,28]. This phenomenon can detach gene family member-specific variants from their host family member, thereby contributing to the variability of the variant ratio at a SFV position across a population. Partial deletion and partial duplication samples are then subtyped utilizing the markers' newly derived applicability. Finally, the concordance of data pointing to the assigned subtypes is evaluated to check the probability of the conclusions.

The four DAZ genes were chosen as the targets of variant ratio analysis, but it can be extended to other gene families. The studied SFV positions were selected by aligning the sequences of the four DAZ family members extracted from the human reference genome. Overall, this integrated method is sufficient for grouping samples according to partial rearrangement along with subtyping deletion and duplication samples. In this paper, we describe the development of variant ratio analysis, and discuss the aspects of its applications and possible extensions.

## Methods

### Collecting genomic DNA

In addition to DNA obtained from the blood of healthy volunteers, germline DNA samples of several TGCT patients with documented gr/gr or b1/b3 deletion were also analyzed in order to enrich for AZFc rearrangements. Altogether, 52 samples were used for this study. All DNA donors gave their informed written consent to the procedure described in this paper. The use of clinical material was approved by the Institutional Review Board of the National Institute of Oncology. (Name of EC: ETT TUKEB, approval number: 20998-0/2010-1018EKU (845/PI/010).)

### Amplification and sequencing of individual samples

**Fig 2:**
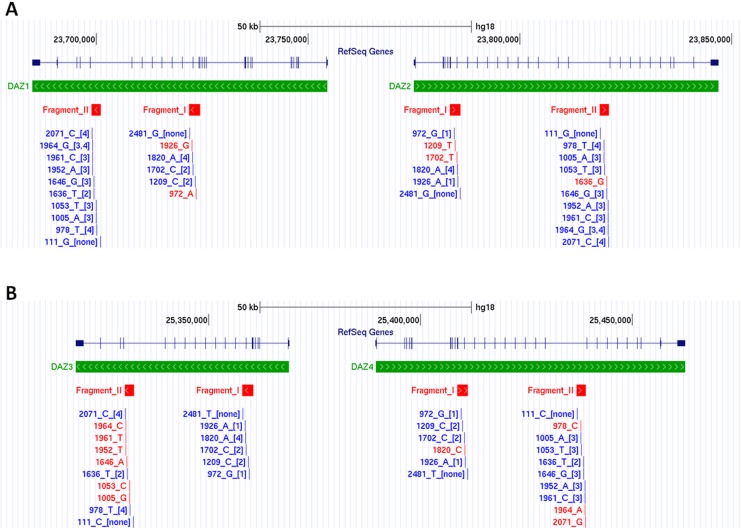
Schematic representation of the four DAZ family members along with the studied markers. Panel A: DAZ1 and DAZ2. Panel B: DAZ3 and DAZ4. The figure was captured from the UCSC Genome Browser after uploading an appropriate BED file (S7 File). Below the scale, chromosome Y coordinates according to the human genome build NCBI36/hg18 are shown. Below the RefSeq Genes label, the structure of the DAZ family members are illustrated with the exons indicated by perpendicular lines. The aligned arrowheads in the thick green line show the direction of the coding sequence. Variants presented in red and blue colors are specific and non-specific for the corresponding DAZ family members, respectively. For non-specific variants, a digit in square brackets indicates the DAZ family member for which the relevant SFV position contains a specific variant. The numbers in the variants' name indicate the variants' location in Fragments I or II.

After aligning the sequences of the four DAZ family members using Multalin sequence alignment tool [29], two regions were selected for amplification. The products were designated Fragments I and II (Fig 2). Both were designed to consist of four amplicons derived from the four DAZ family members, respectively, and to contain several SFV positions (S1 Fig, upper part; S1 and S2 Tables; S5–S7 Files). Fragments I and II were amplified in 52 individual samples using the Qiagen Multiplex PCR Kit. In order to characterize all SFV positions, both fragments were sequenced with four sequencing primers on an ABI 3130 Genetic Analyzer using the Big Dye Terminator v1.1 Cycle Sequencing Kit. Both kits were used according to the manufacturer’s instructions. The sequences of all primers utilized in this study are shown in S3 Table.

### Preparation of control DNA mixtures

After inspection of the sequencing electropherograms, two samples were selected that contained every DAZ family member-specific SFV in the region covered by Fragment I. The fragment was freshly amplified and cloned with Invitrogen’s TOPO TA Cloning Kit as described in the manufacturers’ protocol. For either sample, overnight cultures were grown from 30 white colonies. Plasmids were cleaned up using Invitrogen’s PureLink Plasmid Miniprep Kit. The concentration of the plasmid preparations was measured and adjusted to 100.0 ng/μl. The inserts of the preparations were sequenced to find out which DAZ family member they must have been derived from. Four plasmid preps with insert of different origins were chosen to assemble control DNA mixtures in order to mimic Fragment I amplified in samples containing two, four or six DAZ copies in various combinations. The mixtures presented several known variant ratios at every SFV position. The composition of the DNA mixtures is shown in S4 Table.

### Analysis of control DNA mixtures

To adjust the concentration of the DAZ inserts to the value typical of DAZ genes in individual DNA samples in our lab, the control mixtures were diluted 1:500,000. Six aliquots of mixture 1 and three aliquots of mixtures 2–9 were amplified parallel and sequenced as described above. The area-under-the-curve (AUC) ratio was determined from the sequencing electropherograms at each studied SFV position in each aliquot of every DNA mixture. The AUC of the signal given by the DAZ family member-specific variant (AUC_spec_) and that given by the non-specific variant (AUC_non-spec_) was measured separately with the use of ImageJ software [30] in a standardized way. The AUC ratio was calculated using the following formula: 100 x AUC_spec_/(AUC_spec_ + AUC_non-spec_). An average AUC ratio ± SD was determined and assigned to each known variant ratio at any tested SFV position (S5 Table).

### Analysis of individuals’ samples

**Fig 3:**
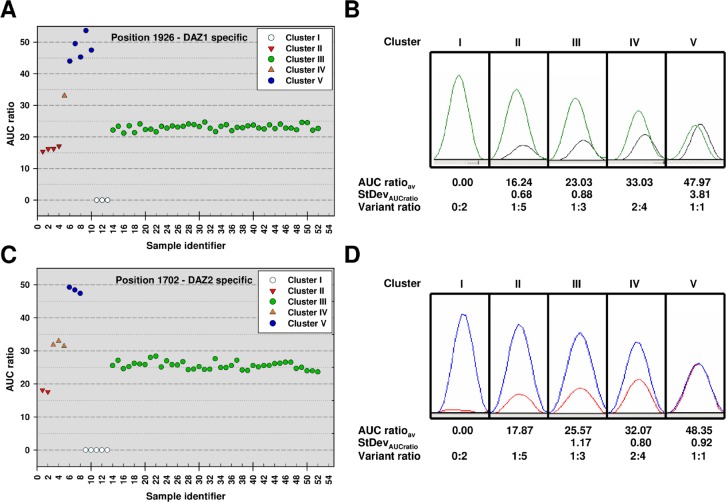
Clustering of AUC ratios at two SFV positions in fifty-two samples. The fifty-two samples sequenced form five distinct clusters according to the AUC ratio measured at SVF positions 1926 (**A**) and 1702 (**C**). Samples 1–5 are the duplication samples (referred to as Ydup_01–05 in the text), whereas samples 6–13 are the deletion samples (referred to as Ydel_06–13). Samples 14–52, each having one copy of all four DAZ family members, constitute the control panel. Representative electropherogram pictures of SVF positions 1926 (**B**) and 1702 (**D**) obtained in samples belonging to the distinct clusters are shown. The average (AUC ratio_av_) and standard deviation (StDev) of the AUC ratio values were calculated from all samples belonging to a cluster. Comparing with the AUC ratio–variant ratio relationship determined in control mixes, a variant ratio was assigned to each cluster at both positions. The AUC ratio (presented here as percentage) calculation is described in the Methods section.

The AUC ratio was determined at each SFV position in the 52 samples based on the sequencing electropherograms as described above. The AUC ratios obtained at a given SFV position throughout all samples were compared to one another (Fig 3). Based on cluster formation and, in the case of Fragment I, comparison with the results obtained for the control mixes, too, a variant ratio was assigned to each SFV position in every individual sample. The exact variant ratio for 0:2x, 2x:0 and x:x positions were established after dividing the samples into partial deletion, partial duplication and non-rearranged (control) groups on the basis of their horizontal variant ratio distribution (Table 1).

**Table 1:** Relationship between a sample’s AZFc partial deletion/duplication status and its horizontal variant ratio distribution.

AZFc partial deletion/duplication status	Type of SFV positions (#specific variant: #non-specific variant)						
	0:2x	2x:0	x:x	1:3	1:5	2:4	4:2
No partial rearrangement (x = 2)	+	-	+	+	-	-	-
Partial deletion affecting two DAZ family members (x = 1)	+	+	+	-	-	-	-
Partial deletion affecting two DAZ family members followed by duplication (x = 2)	+	+	+	-	-	-	-
Partial duplication affecting two DAZ family members (x = 3)	+	-	+	-	+	+	+

A + sign means that the corresponding type of SFV position may be present in a sample with the relevant deletion/duplication status. A - sign means that the corresponding type of SFV position is not expected in a sample with the relevant deletion/duplication status. The value of x is the function of the deletion/duplication status.

### Validation methods

#### Multiplex PCR assays to detect deletions in AZFc region

Two multiplex PCR reactions were assembled to amplify three and four STS markers, respectively, which are located in AZFc region (except for sY1201). Multiplex I amplified sY1197 (NCBI GenBank Accession Number: G67168), sY1206 (G67171) and sY1201 (G67170). Multiplex II tested for the presence of sY1191 (G73809), sY1291 (G72340), sY1258 (G75499) and sY1201. Lying outside the AZFc region, sY1201 was used as a control for the presence of chromosome Y and the functionality of the PCR reaction in both multiplex assemblies. The reactions were carried out using the Qiagen Multiplex PCR Kit according to the manufacturer’s instructions. The ratios of the primers were optimized so that each band would exhibit approximately equal intensity on agarose gels.

#### Gene dosage test for the DAZ gene family

The cumulative copy number of the DAZ gene family was validated by a previously described method [31]. Briefly, the target (DAZ) and an internal control with known copy number (DAZL) were co-amplified using a FAM-labelled primer pair. The amplification was stopped in the exponential phase. The DAZ and DAZL fragments, which had a 3 bp difference in length, were separated by polyacrylamide gel electrophoresis on an ABI 310 Genetic Analyzer. The quantification was performed by comparing the peak area of the target to that of the internal control.

#### Cloning and sequencing selected samples to study the co-segregation characteristics of DAZ family member-specific variants

Fragments I and II were amplified and cloned, as described above, in four samples selected so that as many SFV positions as possible show a variant ratio of 1:3. In the case of each cloned fragment, the insert was amplified in colony PCR from 30 white colonies using KAPA2G Robust HotStart DNA Polymerase. The products were sequenced and consensus variant series thought to represent the different DAZ family members were determined for both fragments in all four samples.

## Results

### Evaluation of the control DNA mixtures

#### Determination of the variant ratio—AUC ratio relationship

**Fig 4:**
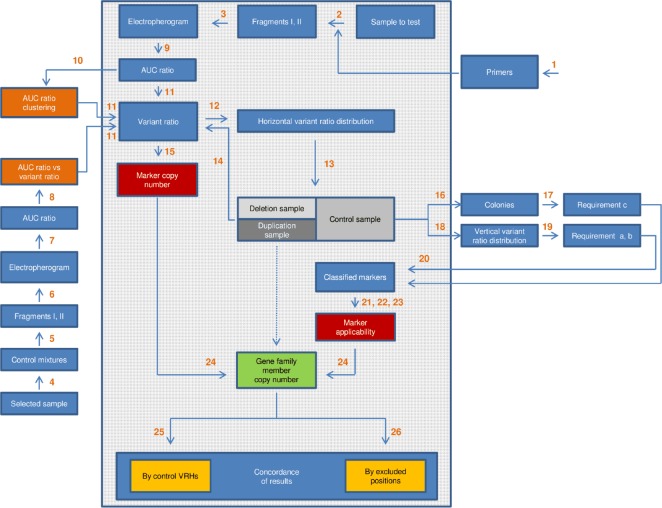
Flowchart showing the steps involved in the elaboration and application of variant ratio analysis. The numbered arrows stand for the processes applied, the rectangles symbolize the result of the respective process. The green rectangle shows the desired end-result of the analysis. Brown rectangles symbolize calibration data used to help derive variant ratios from AUC ratios. Red rectangles stand for input data required for stage 2 or, if there are marker associations, stage 3 analysis. Samples with different partial rearrangement types are indicated by three different shades of gray. The dashed arrow means that only deletion and duplication samples undergo stage 2 or stage 3 analysis. The large rectangle with transparent grey background contains the steps that are required for a complete analysis. The steps outside the large rectangle were used for the elaboration of the method. All processes are listed below. 1/ Aligning the sequences of the four DAZ genes extracted from the human reference genome in order to select amplifiable fragments which i) consist of four amplicons belonging to the four DAZ genes, respectively, and ii) contain as many SFV positions as possible (Fragments I and II) (Fig 2, S1 Fig, S5–S7 Files, S1 and S2 Tables). 2/ Amplification of selected regions in all individual samples to get Fragments I and II. 3/ Sequencing Fragments I and II in all individual samples. 4/ Preparing control plasmid mixtures by cloning Fragment I amplified from samples selected to contain every DAZ1, DAZ2, DAZ3 and DAZ4-specific variant in order to mimic wild-type, AZFc partial deletion and AZFc partial duplication samples having *known* variant ratios at each SFV position (S4 Table). 5/ Amplification of selected regions to get Fragment I in control mixtures. 6/ Sequencing Fragment I in control mixtures. 7/ Measuring AUCs by ImageJ software and calculating the AUC ratio at each SFV position in every control mixture. 8/ Correlating the AUC ratios measured in control mixtures with known variant ratios (S5 Table). 9/ Measuring AUCs and calculating the AUC ratio at each SFV position in all individual samples. 10/ Plotting AUC ratios throughout all samples for each studied position to visualize AUC ratio clustering (Fig 3). 11/ Assigning a variant ratio to each SFV position in all individual samples. 12/ Determining the horizontal variant ratio distribution in all individual samples. 13/ Grouping samples according to AZFc partial deletion/duplication status based on their horizontal variant ratio distribution (Tables 1–4). The results of this step were validated by a generally accepted DAZ dosage test (not shown) and two multiplex PCRs amplifying six sY markers (S2 Fig). 14/ Specifying the variant ratios that remained ambiguous on the basis of the AUC ratio (electropherogram picture) in step 11 (0:2x, x:x and 2x:0 positions). 15/ Deducing the copy number of the specific variant at each SFV position in all individual samples from the relevant variant ratio. 16/ Cloning Fragments I and II in four selected control samples to separate the four amplicons derived from the four DAZ family members, respectively. 17/ Sequencing an appropriate number of colonies in order to study the co-segregation of DAZ family member-specific variants, i.e. the fulfillment of requirement (c) imposed on an ideal marker (S6 Table).18/ Determining the vertical variant ratio distribution throughout all control samples at each studied SFV position. 19/ Determining p1 and p2 values on the basis of the vertical variant ratio distribution at each SFV position within the control panel in order to study the fulfillment of requirements (a) and (b) also imposed on an ideal marker (Table 4, the equations are seen in the text). 20/ Classifying family member-specific variants on the basis of p1 and p2, which results in the distinction of class I, class II/a, class II/b and class III markers (Table 5). 21/ Determining the relationship between the copy number of a gene family member-specific variant and the copy number of the corresponding gene family member for class II/a and class II/b markers. It results in the “restricted” applicability of the markers that will be utilized at stage 2 and stage 3 analyses (S1 and S2 Files, Tables 6 and 7). 22/ Searching for perfect associations between variants specific to different DAZ family members in the control panel in order to re-evaluate (extend) the applicability of some markers if possible (Table 4). 23/ Determining the relationship between the copy number of the members of the associated marker pairs and the copy number of the corresponding gene family members for the relevant class II/a and class II/b markers, which results in their “extended” applicability that will be utilized at stage 3 analysis (S3 and S4 Files, Tables 8 and 9). 24/ Evaluating partial deletion and partial duplication samples identified in step 13 using the classified markers with restricted applicability (stage 2 and 3) or, if exists, extended applicability (stage 3) to determine the samples’ deletion and duplication subtype (S8 and S9 Tables, Tables 2 and 3). 25/ Comparing the series of the variant ratios (variant ratio haplotype, VRH) of the deletion and duplication samples with the VRHs observed in the control panel in order to support the existence of the assigned rearrangement subtypes in the studied population (S7 and S11 Tables). 26/ Checking if the assigned rearrangement subtypes are concordant with or might even be indicated by the variant ratios determined at positions also examined but not utilized for stage 2 and stage 3 analyses for some reason (S10 Table).

The steps involved in either the elaboration or the application of variant ratio analysis are depicted by a flowchart shown by Fig 4.

Several control DNA mixtures having known variant ratios at the studied SFV positions were prepared to mimic various deletions and duplications affecting Fragment I. The AUC ratios measured in them well reflected the variant ratios at any selected SFV position. DNA mixtures with identical variant ratio at a given SFV position formed a distinct cluster according to their AUC ratio at the relevant position. Several such clusters could be distinguished at any SFV position. The AUC ratios of the members of a cluster were averaged and the average ± SD was assigned to the respective variant ratio at the given SFV position. As an example, the data calculated at SFV positions 1702 and 1926 in Fragment I are shown in S5 Table. This relationship can be used to determine the variant ratio from the measured AUC ratio at the relevant SFV position in the analysis of individual samples.

### Evaluation of individual DNA samples

#### Determination of the AZFc partial deletion/duplication status of samples and the copy number of the family member-specific variants

After calculating the AUC ratio, a variant ratio was assigned to every single SFV position selected for analysis in all of the 52 individual samples. It was achieved partly on the basis of the AUC ratio–variant ratio relationship determined by the analysis of control mixtures (at SFV positions in Fragment I), partly by ordering the clusters formed by the samples according to their AUC ratio (at SFV positions in Fragments I and II) (Fig 3). Cluster formation in itself seemed to be sufficient for variant ratio assignment. In the next step, using the theoretical relationship presented and explained in Table 1, the AZFc partial deletion/duplication status of the samples was inferred from their “horizontal” variant ratio distribution. Eight and five samples were concluded to carry deletion (0:2x, 2x:0 and x:x type positions only, Table 2) or duplication (0:2x, x:x, 1:5, 2:4 and 4:2 type positions, Table 3), respectively. These findings were verified by two independent, previously validated methods. First, an STS marker analysis by two multiplex PCR assays showed that only eight samples found by variant ratio analysis to carry deletion lacked one or three of the tested STS markers (S2 Fig). Second, the DAZ dosage test [31] confirmed the cumulative DAZ copy number in all deletion and duplication samples identified by variant ratio analysis. Since x takes the value of 1, 2 and 3 in partial deletion, control and partial duplication samples, respectively, the variant ratios of 0:2x, 2x:0 and x:x positions were changed to 0:2, 2:0 and 1:1 in deletion samples, 0:4 and 2:2 in control samples and 0:6 and 3:3 in duplication samples (there was no 2x:0 position among the controls and the duplication samples). Based on the variant ratios, the copy number of the specific variant(s) was determined at each SFV position (S8A and S9A Tables).

**Table 2:** The human reference genome-based characteristics of the studied SFV positions and their variant ratios found in eight partial deletion samples.

	SFV position on Fragment I						SFV position on Fragment II											Deleted	Identifier
Location	972	1209	1702	1820	1926	2481^2^	111^3^	978	1005	1053	1636	1646	1952	1961	1964^4^	1964^4^	2071		
Marker specificity	DAZ1	DAZ2	DAZ2	DAZ4	DAZ1	-	-	DAZ4	DAZ3	DAZ3	DAZ2	DAZ3	DAZ3	DAZ3	DAZ3	DAZ4	DAZ4		
Variants^1^	A:G	T:C	T:C	C:A	G:A	G:T	G:C(:T)	C:T	G:A	C:T	G:T	A:G	T:A	T:C	C:(A+G)	A:(C+G)	G:C		
Variant ratios	0:2	1:1	1:1	0:2	1:1	1:1	1:1^5^	0:2	0:2	0:2	1:1	0:2	0:2	0:2	0:(0+2)	0:(0+2)	0:2	DAZ3/4	Ydel_06
	0:2	1:1	1:1	0:2	1:1	2:0	2:0	0:2	0:2	0:2	1:1	0:2	0:2	0:2	0:(0+2)	0:(0+2)	0:2	DAZ3/4	Ydel_08
	1:1	1:1	1:1	0:2	1:1	2:0	2:0	0:2	0:2	0:2	0:2^5^	0:2	0:2	0:2	0:(0+2)	0:(0+2)	0:2	DAZ3/4	Ydel_07
	1:1	0:2	0:2	0:2	1:1	1:1	1:1	0:2	1:1	1:1	0:2	1:1	1:1	1:1	1:(0+1)	0:(1+1)	0:2	DAZ2/4	Ydel_09
	1:1	0:2	0:2	0:2	1:1	1:1	1:1	0:2	1:1	1:1	0:2	1:1	1:1	1:1	1:(0+1)	0:(1+1)	0:2	DAZ2/4	Ydel_10
	0:2	0:2	0:2	1:1	0:2	0:2	0:2	0:2	2:0	2:0	0:2	2:0	2:0	2:0	2:(0+0)	0:(2+0)	0:2	DAZ1/2	Ydel_11
	0:2	0:2	0:2	1:1	0:2	0:2	0:2	0:2	2:0	2:0	0:2	2:0	2:0	2:0	2:(0+0)	0:(2+0)	0:2	DAZ1/2	Ydel_12
	0:2	0:2	0:2	1:1	0:2	0:2	0:2	0:2	2:0	2:0	0:2	2:0	2:0	2:0	2:(0+0)	0:(2+0)	0:2	DAZ1/2	Ydel_13

^1^Variants are arranged as family member-specific variant(s):non-specific variant(s) at each SFV position.

^2^At position 2481 in Fragment I, there is no specific variant (DAZ1/2: G, DAZ3/4: T according to the human reference assembly).

^3^At position 111 in Fragment II, there is no specific variant (DAZ1/2: G, DAZ3/4: C according to the human reference assembly). In several of our samples, a T is substituted for one of the Cs.

^4^At position 1964, there is a DAZ3-specific C and a DAZ4-specific A according to the human reference assembly.

^5^Variant ratio not in accordance with the concluded deletion subtype

**Table 3:** The human reference genome-based characteristics of the studied SFV positions and their variant ratios found in five partial duplication samples.

	SFV position on Fragment I						SFV position on Fragment II											Duplicated	Identifier
Location	972	1209	1702	1820	1926	2481^2^	111^3^	978	1005	1053	1636	1646	1952	1961	1964^4^	1964^4^	2071		
Marker specificity	DAZ1	DAZ2	DAZ2	DAZ4	DAZ1	-	-	DAZ4	DAZ3	DAZ3	DAZ2	DAZ3	DAZ3	DAZ3	DAZ3	DAZ4	DAZ4		
Variants^1^	A:G	T:C	T:C	C:A	G:A	G:T	G:C(:T)	C:T	G:A	C:T	G:T	A:G	T:A	T:C	C:(A+G)	A:(C+G)	G:C		
Variant ratios	0:6	1:5	1:5	0:6	1:5	2:4	2:4	1:5	3:3	3:3	1:5	3:3	3:3	3:3	3:(0+3)	0:(3+3)	1:5	DAZ3/4	Ydup_01
	0:6	2:4	2:4	0:6	2:4	2:4	4:2	1:5	1:5	1:5	2:4	1:5	1:5	1:5	1:(0+5)	0:(1+5)	1:5	DAZ1/2	Ydup_05
	1:5	2:4	2:4	2:4	1:5	1:5	3:3	0:6	3:3	3:3	2:4	3:3	3:3	3:3	3:(0+3)	0:(3+3)	0:6	DAZ2/4	Ydup_03
	1:5	2:4	2:4	2:4	1:5	1:5	3:3	0:6	3:3	3:3	2:4	3:3	3:3	3:3	3:(0+3)	0:(3+3)	0:6	DAZ2/4	Ydup_04
	0:6	1:5	1:5	0:6	1:5	1:5	2:4	2:4	2:4	2:4	1:5	2:4	2:4	2:4	2:(2+2)	2:(2+2)	2:4	DAZ3/4	Ydup_02

^1^Variants are arranged as family member-specific variant(s):non-specific variant(s) at each SFV position.

^2^At position 2481 in Fragment I, there is no specific variant (DAZ1/2: G, DAZ3/4: T according to the human reference assembly).

^3^At position 111 in Fragment II, there is no specific variant (DAZ1/2: G, DAZ3/4: C according to the human reference assembly). In several of our samples, a T is substituted for one of the Cs.

^4^At position 1964, there is a DAZ3-specific C and a DAZ4-specific A according to the human reference assembly.

Supposing pairwise deletion and duplication of the DAZ family members, one of seven different variant ratios (0:2x, 2x:0, x:x, 1:3, 1:5, 2:4 and 4:2) can be assigned to an SFV position on the basis of its electropherogram picture. Except for 2x:0 and x:x, those ratios directly show the copy number of the family member-specific variant at their respective position. The horizontal variant ratio distribution means the distribution of the different types of SFV positions of a sample. The AZFc partial deletion/duplication status can be determined from the horizontal variant ratio distribution. The electropherogram picture of type 0:2x (0:2, 0:4 or 0:6), type 2x:0 (2:0 or 4:0) and type x:x (1:1, 2:2 or 3:3) sites appears identical, respectively. Their exact variant ratio and, in turn, the copy number of the specific variant at the 2x:0 and x:x type positions can be obtained from the AZFc partial deletion/duplication status of the sample. Subtyping uses both the AZFc partial deletion/duplication status and the copy number of the specific variant(s) at each SFV position as the starting point. [There are positions, such as position 1964 in Fragment II, which comprise more than two variants in certain samples; therefore, their description is necessarily more complex. For example, in the view of DAZ3, the integers in the formula 1:(1+2) mean one specific C, one non-specific A (which, at the same time, is specific to DAZ4) and two non-specific Gs. Overall, it refers to a 1:3 type position. Under the same considerations, 1:(0+3) is identical with 1:3; 0:(2+2) and 0:(1+3) with 0:4; and 2:(0+2) with 2:2.]

Based on variant ratios only, no distinction can be made between partial deletion and partial deletion followed by duplication. Therefore, samples found to carry partial deletion must be checked using a dosage test to determine if they also underwent duplication. In a similar way, the identification of samples with the entire AZFc region duplicated requires to subject non-rearranged samples to a dosage test. However, the lack of knowledge of the exact DAZ copy number of samples belonging to these two categories does not influence subtyping.

#### Selection of samples to construct a control panel for marker classification

Out of the 52 samples tested in this work, 39 showed a horizontal variant ratio distribution that is characteristic of the presence of four different DAZ genes (i.e. 0:2x, x:x and 1:3 type positions only, Table 4). All of them carried SFVs specific to each DAZ family member except one sample that showed no DAZ4 specific variants at all (variant ratio haplotype [VRH] 4). In addition, none of them lacked any of the STS markers according to the two multiplex PCR assays. Therefore, they were all concluded to possess the four DAZ gene family members in 1:1:1:1 ratio. Since these samples were thought not to have been affected by either partial deletion or partial duplication, they were chosen to assess the variability caused by interlocus gene conversion and/or SNPs in the copy number of SFVs designated DAZ family member-specific based on the human reference genome.

**Table 4:** The human reference genome-based characteristics of the studied SFV positions and their variant ratios determined in 39 control samples grouped according to their variant ratio haplotype.

	SFV position on Fragment I						SFV position on Fragment II											VRH^5^	No.^6^
Location	972	1209	1702	1820	1926	2481^2^	111^3^	978	1005	1053	1636	1646	1952	1961	1964^4^	1964^4^	2071		
Marker specificity	DAZ1	DAZ2	DAZ2	DAZ4	DAZ1	-	-	DAZ4	DAZ3	DAZ3	DAZ2	DAZ3	DAZ3	DAZ3	DAZ3	DAZ4	DAZ4		
Variants^1^	A:G	T:C	T:C	C:A	G:A	G:T	G:C(:T)	C:T	G:A	C:T	G:T	A:G	T:A	T:C	C:(A+G)	A:(C+G)	G:C		
Variant ratios	1:3	1:3	1:3	1:3	1:3	2:2	2:2	1:3	1:3	1:3	1:3	1:3	1:3	1:3	1:(1+2)	1:(1+2)	1:3	RefSeq^7^	0
	0:4	1:3	1:3	0:4	1:3	1:3	2:2	1:3	1:3	1:3	1:3	1:3	1:3	1:3	1:(0+3)	0:(1+3)	1:3	3b	12
	0:4	1:3	1:3	0:4	1:3	1:3	2:2	1:3	1:3	1:3	1:3	1:3	1:3	1:3	1:(1+2)	1:(1+2)	1:3	3a/1	2
	0:4	1:3	1:3	0:4	1:3	1:3	2:1(:1)	1:3	1:3	1:3	2:2	1:3	1:3	1:3	1:(1+2)	1:(1+2)	1:3	3a/2	3
	0:4	1:3	1:3	0:4	1:3	2:2	2:2	1:3	1:3	1:3	1:3	1:3	1:3	1:3	1:(1+2)	1:(1+2)	1:3	3a/3	1
	1:3	1:3	1:3	1:3	1:3	2:2	2:2	1:3	1:3	1:3	1:3	1:3	1:3	1:3	1:(0+3)	0:(1+3)	1:3	2	6
	1:3	1:3	1:3	1:3	1:3	1:3	2:2	0:4	2:2	2:2	1:3	2:2	2:2	2:2	2:(0+2)	0:(2+2)	0:4	1	13
	0:4	1:3	1:3	0:4	1:3	2:2	N/A	0:4	0:4	0:4	1:3	2:2	2:2	2:2	2:(0+2)	0:(2+2)	0:4	4	1
	0:4	1:3	1:3	0:4	1:3	2:2	N/A	1:3	0:4	0:4	1:3	1:3	1:3	1:3	1:(0+3)	0:(1+3)	1:3	3c	1
p1^8^	1.00	1.00	1.00	1.00	1.00	-	-	1.00	0.65	0.65	0.92	0.64	0.64	0.64	0.64	1.00	1.00		
p2^8^	0.49	1.00	1.00	0.49	1.00	-	-	0.64	0.92	0.92	1.00	1.00	1.00	1.00	1.00	0.15	0.64		
Class of marker	II/b	I	I	II/b	I	-	-	II/b	III	III	II/a	II/a	II/a	II/a	II/a	II/b	II/b		

^1^Variants are arranged as family member-specific variant(s):non-specific variant(s) at each SFV position.

^2^At position 2481 in Fragment I, there is no specific variant (DAZ1/2: G, DAZ3/4: T according to the human reference assembly).

^3^At position 111 in Fragment II, there is no specific variant (DAZ1/2: G, DAZ3/4: C according to the human reference assembly). In three samples (VRH 3a/2), T was found to replace one of the Cs. Based on cloning experiments, T is located in DAZ3 (S6C Table)

^4^At position 1964, there is a DAZ3-specific C and a DAZ4-specific A according to the human reference assembly.

^5^Variant ratio haplotypes were named arbitrarily based on similarities.

^6^Number of samples belonging to a variant ratio haplotype among the 39 controls

^7^RefSeq variant ratio haplotype was determined by the alignment of the corresponding DAZ regions derived from the human reference assembly hg18.

^8^The meaning and calculation of p1 and p2 can be found in the text

#### Diversity of the variant ratio haplotypes (VRHs) found in the control panel

The series of the successive variant ratios define a sample’s VRH. An important aspect of this study was to get insight into the various VRHs that are present in the Hungarian population, in men who carry one copy of each member of the DAZ gene family. The eight different VRHs distinguishable in the control panel are indicated in Table 4. Not surprisingly, no individual with a VRH identical to the one that can be derived from the human reference genome was found. The great inter-individual variability observed in the variant ratio of a SFV position in general (S1 Fig, lower part and Table 4) could confuse the evaluation of unknown samples during the search for AZFc rearrangement subtypes. Therefore, in order to avoid drawing false conclusions, it proved to be indispensable to establish a marker classification system.

#### Classification of “specific” SFVs based on how they meet requirements imposed on an ideal marker

The purpose of marker classification was to revise the single reference genome-conferred specificity status of SFVs. The revision was carried out on the basis of observations made in a group of non-rearranged (i.e. control) samples.

An ideal DAZ family member-specific marker must fulfill three requirements. It must be present in all samples that contain each one of the four DAZ genes (a); it must not be present in more than one member of the DAZ gene family in any sample (b); the DAZ family member containing the marker must be the same in every sample, which also implies that it must be identical with the one to which the marker was associated by the human reference genome (c). The conclusions allowed to be drawn from the presence or absence of a marker depends on which one(s) of the above requirements it fulfills.

Meeting requirement (c) is a prerequisite for a variant to be utilized as a gene family member-specific marker. In order to study the fulfillment of this condition, the co-segregation characteristics of SFVs were examined in four control samples that had been selected in the knowledge of their VRH. The four DAZ family member-specific amplicons constituting Fragments I and II, respectively, were separated by cloning. The consensus SFV series thought representative of the four DAZ family members, respectively, were determined by sequencing the insert in a sufficient number of colonies (S6 Table). The results were in complete accordance with the samples’ VRH in all cases. In addition, we observed that variants considered specific to a given family member co-segregated in the same amplicon in each sample whenever they were found. On the other hand, variants thought specific to different DAZ genes never showed co-segregation provided that they were present in no more than one family member according to the observed variant ratios. In summary, variants associated to different DAZ genes by the reference genome were really located on distinct family members in all four samples. This led us to the assumption that requirement (c) can be considered to be met *in general* in the case of our studied SFVs.

The “vertical” variant ratio distribution that can be obtained upon the examination of samples belonging to the control panel indicates whether a DAZ family member-specific variant meets requirements (a) and/or (b). Vertical variant ratio distribution means the distribution of the variant ratios that a SFV position shows in a defined group of samples. The expected “bins” of that distribution in the control group are 0:4, 1:3 and 2:2. Two numerical values were computed from that distribution for every SFV according to the following formulas: p1 = subjects_1:3_ / [subjects_1:3_ + subjects_2:2_] and p2 = subjects_1:3_ / [subjects_1:3_ + subjects_0:4_]), where subjects_1:3_, subjects_2:2_ and subjects_0:4_ stand for the number of samples with a variant ratio of 1:3, 2:2 and 0:4, respectively, in a given SFV position throughout all control samples (Table 4). p1 reflects the probability that the DAZ family member, which the given variant is considered to be specific to, is present in a deletion sample in which the variant has been found. p2 indicates the probability that the DAZ family member, which the given variant is considered to be specific to, is absent in a deletion sample in which the lack of the variant has been demonstrated. A cut-off of 0.95 was set for both p1 and p2. DAZ family member-specific SFVs were classified according to the way their p1 and p2 values were related to the cut-off. If p1 and/or p2 exceed the cut-off value, requirements (b) and/or (a) must be considered to be fulfilled, respectively (Table 5).

**Table 5:** Classification of DAZ family member-specific markers.

Evaluation of control samples				Evaluation of unknown samples	
p1	p2	Requirements fulfilled	Class of marker	Inference that could be drawn from the presence of the marker	Inference that could be drawn from the absence of the marker
> cut-off_1_	> cut-off_2_	(a), (b)	I	DAZ family member present	DAZ family member absent
> cut-off_1_	≤ cut-off_2_	(b)	II/b	DAZ family member present	-
≤ cut-off_1_	> cut-off_2_	(a)	II/a	-	DAZ family member absent
≤ cut-off_1_	≤ cut-off_2_	-	III	-	-

Variants have been classified according to how they fulfill requirements (a) and (b) imposed on an ideal family member-specific marker. Cut-off_1_ and cut-off_2_ can be arbitrarily set. p1 and p2 are the reliability values which can be calculated from the vertical variant ratio distribution obtained in the control panel at the respective SFV position (the equations are seen in the text).

#### Finding the relationship between a marker’s copy numbers and the corresponding gene family member’s copy numbers

The aim of variant ratio analysis is to find the relationship between the copy numbers of a gene family member-specific variant and the copy numbers of the corresponding gene family member itself. Marker classification helps this procedure by allowing to establish the above relationship for each marker class, separately. The copy number of a gene family member can be changed by a large genomic rearrangement, i.e. deletion or duplication. However, the copy number of a specific variant is also affected by local changes such as gene conversion. It means that, as opposed to class I markers, class II/a, class II/b and class III markers are detached from the gene family member they are supposed to be specific to.

The copy number of a class I marker always directly indicates the copy number of its carrier DAZ family member. The same does not hold true of class II/a and class II/b markers, which is shown by the findings that they were present with 2 and 0 copies, respectively, in several control samples. In order to determine the copy numbers of a marker that unambiguously indicate the copy number of the corresponding gene family member, we first determined the whole spectrum of gene conversions assumed to be capable of duplicating a given class II/a or eliminating a given class II/b marker, respectively. Then, each conversion was combined with all theoretically possible DAZ deletion and duplication subtypes (S1 and S2 Files). The informative copy numbers of a marker overall, that can be obtained by the marker’s separate (i.e. association-independent) evaluation, is called its restricted applicability (Tables 6 and 7). Since the applicability of class III markers is very limited, only class I, class II/a and class II/b markers were kept for the analysis of partial deletion and partial duplication samples (S8 File).

**Table 6:** Restricted applicability of class II/a markers.

Copy number of a class II/a marker	Copy number of the DAZ family member	
	Deletion samples	Duplication samples
0	0	-
1	no conclusion	1
2	1	no conclusion
3	-	no conclusion
4	-	2

Conclusions allowed to be drawn from the copy number of a class II/a DAZ family member-specific variant for the copy number of the relevant DAZ family member in partial deletion and partial duplication samples, respectively, are shown. The conclusions were established by determining the whole spectrum of gene conversions assumed to be able to duplicate a DAZ family member-specific variant, then combining each potential pairwise DAZ deletion or duplication event with each gene conversion, the latter allowed to take place either before or after the large rearrangement (S1 File). These conclusions are exploited in the stage 2 and stage 3 analyses of unknown samples (S8 and S9 Tables b-c). The '-' sign means a not expected scenario.

**Table 7:** Restricted applicability of class II/b markers.

Copy number of a class II/b marker	Copy number of the DAZ family member	
	Deletion samples	Duplication samples
0	no conclusion	no conclusion
1	1	no conclusion
2	-	2

Conclusions allowed to be drawn from the copy number of a class II/b DAZ family member-specific variant for the copy number of the relevant DAZ family member in partial deletion and partial duplication samples, respectively, are shown. The above conclusions were established by determining the whole spectrum of gene conversions assumed to be able to eliminate a DAZ family member-specific variant, then combining each potential pairwise DAZ deletion or duplication event with each gene conversion, the latter allowed to take place either before or after the large rearrangement (S2 File). These conclusions are exploited in the stage 2 and stage 3 analyses of unknown samples (S8 and S9 Tables b-c). The '-' sign means a not expected scenario.

#### Alleviating the classification-imposed restrictions on the applicability of certain class II/a and class II/b markers

By definition, the utilization of class II/a and class II/b markers is restricted to one direction, *i*.*e*. the presence of the former- and the absence of the latter-category-markers is considered to be non-informative. However, this restricted applicability may be modified by the *combined* evaluation of markers that are specific to different family members and, at the same time, show association in the control panel. Two such associations could be discovered in our data set (Table 4).

First, the class II/b DAZ1-specific “A” at position 972 (A_972_) and the class II/b DAZ4-specific “C” at position 1820 (C_1820_), both located in Fragment I, were found to be in perfect linkage disequilibrium. As a result, the presence of one of them endows the otherwise non-informative absence of the other with significance. For example, the absence of the DAZ1-specific A_972_ does not suggest the lack of DAZ1 in a deletion sample in itself. However, in the presence of the DAZ4-specific C_1820_, the absence of the DAZ1-specific A_972_ is supposed to be due to something other than gene conversion, thereby indicating the lack of DAZ1.

Second, the disappearance of class II/b DAZ4-specific variants (C_978_ and G_2071_) always coincided with the duplication of class II/a DAZ3-specific ones (A_1646_, T_1952_, T_1961_ and C_1964_) in Fragment II. We supposed that DAZ3>DAZ4 gene conversion was responsible for all those changes. On one hand, if the DAZ4-specific variants are present, the DAZ3-specific variants, if also present, must be located on DAZ3. Consequently, the concomitant presence of the class II/b DAZ4-specific variants makes the presence of the class II/a DAZ3-specific ones a reliable indicator of the presence of DAZ3. On the other hand, if the DAZ3-specific variants are absent, the DAZ4-specific variants, if also absent, could not have been removed by gene conversion. Therefore, the concomitant absence of the DAZ3-specific variants makes the absence of the DAZ4-specific ones a reliable indicator of DAZ4 deletion.

In order to determine the copy numbers of the participant markers that unambiguously indicate the copy number of the corresponding gene family members, we first determined the whole spectrum of gene conversions or conversion pairs assumed to be capable of producing simultaneously both components of the above associations. Then, each conversion was combined with all theoretically possible DAZ deletion and duplication subtypes (S3 and S4 Files). The informative copy numbers of a marker overall, that can be obtained by an investigation taking the associations of the marker into consideration, is called its extended applicability (Tables 8 and 9).

**Table 8:** Extended applicability of DAZ1-specific A_972_ and DAZ4-specific C_1820_.

Copy number of specific variants		Copy number of DAZ family members			
		Deletion samples		Duplication samples	
DAZ1-specific A_972_	DAZ4-specific C_1820_	DAZ1	DAZ4	DAZ1	DAZ4
0	0	no conclusion	no conclusion	no conclusion	no conclusion
0	1	0	1	1	2
1	0	1	0	2	1
1	1	1	1	no conclusion	no conclusion
1	2	-	-	1	2
2	1	-	-	2	1
2	2	-	-	2	2

Conclusions allowed to be drawn for the copy number of DAZ1 and DAZ4 in partial deletion and partial duplication samples, based on the combined analysis of the class II/b DAZ1-specific A_972_ and DAZ4-specific C_1820_, are shown. To establish the above conclusions, the whole spectrum of gene conversions assumed to be able to eliminate the class II/b DAZ1-specific A_972_ and the class II/b DAZ4-specific C_1820_ in Fragment I, jointly or separately but simultaneously, was determined. Based on the perfect association of these two markers, which had been observed in the control panel, that spectrum could be limited to three conversion pairs (DAZ1>DAZ4 plus DAZ4>DAZ1, DAZ3>DAZ4 plus DAZ2>DAZ1and DAZ2>DAZ4 plus DAZ3>DAZ1). The conclusions were derived by combining each potential pairwise DAZ deletion or duplication subtype with each of the three gene conversion pairs, the latter allowed to take place either before or after the large rearrangement (S3 File). These results are exploited in the stage 3 analysis of unknown samples (S8C and S9C Tables). The '-' sign means a not expected scenario.

**Table 9:** Extended applicability of the class II/a DAZ3- and class II/b DAZ4-specific markers located in Fragment II.

Copy number of specific variants		Copy number of DAZ family members			
		Deletion samples		Duplication samples	
DAZ3-specific variants	DAZ4-specific variants	DAZ3	DAZ4	DAZ3	DAZ4
0	0	0	0	-	-
0	1	0	1	-	-
1	0	no conclusion	no conclusion	-	-
1	1	1	1	1	1
2	0	1	1	1	1
2	1	-	-	no conclusion	no conclusion
3	0	-	-	no conclusion	no conclusion
1	2	-	-	1	2
2	2	-	-	2	2
4	0	-	-	2	2
3	1	-	-	2	2

Conclusions allowed to be drawn for the copy number of DAZ3 and DAZ4 in partial deletion and partial duplication samples, based on the combined analysis of the class II/a DAZ3- and class II/b DAZ4-specific markers located in Fragment II, are shown. To establish the above conclusions, the whole spectrum of gene conversions assumed to be able to duplicate the class II/a DAZ3-specific variants and eliminate the class II/b DAZ4-specific variants in Fragment II, jointly or separately but simultaneously, was determined. Based on the perfect association between the duplication of these DAZ3-specific and the lack of these DAZ4-specific markers, which had been observed in the control panel, that spectrum could be limited to DAZ3>DAZ4 gene conversion. The conclusions were derived by combining each potential pairwise DAZ deletion or duplication with DAZ3>DAZ4 gene conversion, the latter allowed to take place either before or after the large rearrangement (S4 File). These results are exploited in the stage 3 analysis of unknown samples (S8C and S9C Tables). The '-' sign means a not expected scenario.

#### Demonstrating the advantages of using the revised applicability of markers

Based on the results of the applicability revision detailed above, gene family member-specific variants can be utilized in three different ways. An analysis that uses SFVs in the traditional manner, as if all of the specific variants were of class I, is called here stage 1 analysis. In that case, one copy of the marker is considered equivalent to one copy of the corresponding gene family member. At the same time, stage 2 and stage 3 analyses apply the revised applicability. At stage 2, the restricted applicability is used for all markers. At stage 3, the extended applicability is exploited for those taking part in a revealed association, whereas the restricted applicability is applied for all others.

We compared the outcome of the three analysis types using our data. At stage 1, we frequently ran against controversy between findings related to a given family member, which resulted, at best, in uncertainty regarding the concluded deletion or duplication subtypes (S8A and S9A Tables). Stage 2 evaluation resulted in the disappearance of the above inconsistencies due to the exclusion of unreliable findings from the analysis (S8B and S9B Tables). It turned out to be sufficient for the successful evaluation of three deletion samples (Ydel_11, Ydel_12 and Ydel_13). However, this improvement often took place at the expense of losing too many data for certain family members to retain the ability to draw any conclusion for their copy number. For example, no exploitable information remained for DAZ4 in the other five deletion and two duplication samples (Ydup_01, Ydup_05). In contrast, stage 3 analysis led to the successful evaluation of our all deletion and duplication samples by making it possible to retain a considerable part of the data which would otherwise have been excluded from the analysis at stage 2 (S8C and S9C Tables). It was the first association which allowed to infer the lack of DAZ4 from the absence of the DAZ4-specific C_1820_ in the concomitant presence of the DAZ1-specific A_972_ in Ydel_07, Ydel_09 and Ydel_10. At the same time, the second association made it possible that reliable copy number data could be obtained for DAZ4 in samples Ydel_06, Ydel_07, Ydel_08, Ydup_01 and Ydup_05 and for DAZ3 in Ydup_01 and Ydup_02.

Preferably, the copy number should be determined for all DAZ family members separately, i.e. independently of the copy number of the other DAZ genes. In this point, we could assign a copy number to 48 out of the 52 (92.31%) DAZ family members in our 13 partially rearranged samples at stage 3 analysis. In four samples (Ydel_09, Ydel_10, Ydup_03, Ydup_04), the DAZ3 copy number was still ambiguous.

#### Summary of the results of subtyping

Our deletion and duplication samples were evaluated by subjecting them to stage 3 analysis in order to determine the samples’ deletion or duplication subtype (i.e. which DAZ family members were deleted or duplicated). The results are shown in Tables 2 and 3. Three different subtypes were identified among the 8 deletion samples: DAZ1/DAZ2, DAZ2/DAZ4 and DAZ3/DAZ4 deletions. The subtypes concluded for the duplication samples showed similar variability: DAZ1/DAZ2, DAZ2/DAZ4 and DAZ3/DAZ4 duplications could be distinguished among the 5 individuals inferred to possess six DAZ copies.

### Possible ways to support the conclusions

#### Calculation of concordance

Positions that carry class I, class II/a or class II/b markers are exploited to assign a rearrangement subtype to a sample. They make up the assignment set of SFV positions. As opposed, the information offered by SFVs not benefited in stage 2 or 3 analysis can be used to check the correctness of the concluded subtype. Those positions were either precluded because they contained no variant specific to a DAZ family member according to the reference genome (position 2481 in Fragment I and position 111 in Fragment II), or excluded upon marker classification since their specific variant was found to belong to class III (positions 1005 and 1053 in Fragment II). They can be utilized as the validation set of SFV positions (S10 Table). The variant ratio of an SFV position can fall into one of three categories. It may (1) unambiguously indicate the assigned subtype by itself; (2) behave neutrally, i.e. neither contradict nor unambiguously indicate the assigned subtype; (3) contradict the assigned subtype. Variant ratios belonging to the 1st or 2nd categories are considered to be concordant with the assignments.

The assignment set covered 12 positions. Out of 169 variant ratios (13 observations in each of 8 deletion and 5 duplication samples), 118 (69.82%) was kept at stage 3 analysis. Out of them, 55 (46.61%), 62 (52.54%) and 1 (0.85%) fell in the indicative, neutral and contradictory category, respectively. The validation set encompassed 4 positions. Out of the 52 observations, only 19 (36.54%) proved to be meaningful with respect to checking the rearrangement status. Out of them, 16 (84.21%), 3 (15.79) and 0 (0.00%) were indicative of, neutral to and contradictory to the assigned subtypes, respectively. The concordance for the assignment and validation sets is 99.15% (117/118) and 100.00% (19/19), respectively. Altogether, 137 out of 221 (61.99%) copy number data were kept for analysis. Out of the 137, 71 (51.82%), 65 (47.45%) and 1 (0.73%) proved to be indicative, neutral and contradictory, respectively. All but one (136, 99.27%) observations were found to be in accordance with the assigned subtypes (Tables 2 and 3).

#### Comparison of the VRH of the deletion and duplication samples with the VRHs observed in the control panel

In six out of eight deletion and four out of five duplication samples, the VRH we found could be explained by the deduced type of rearrangement without any discordance if one of the VRHs observed in the control panel was assumed to be the starting point of the rearrangement (S7 Table). In other words, there was at least one VRH identified in the control population whose rearrangement by the deduced deletion or duplication subtype would result in exactly the same VRH that we observed in the respective deletion or duplication sample. It may mean that the vast majority of the sequence variations as well as their combinations typical of the Hungarian population in the regions corresponding to Fragments I and II are encompassed by our control panel in spite of the relatively low number of individuals it enrolled. The remaining two deletions and one duplication might have occurred on backgrounds with VRHs not represented by our control samples. Another possibility for their existence is that a gene conversion event took place after the deletion or duplication, which modified the VRH produced by the large rearrangement. For example, the VRH of sample Ydup_01 can be explained by supposing that an initial DAZ3/DAZ4 duplication on a background with a VRH different at position 2481 from 3b (2:2 instead of 1:3) *or* at position 1964 from 3a/3 (1:3 instead of 1:1:2) was followed by a gene conversion in one DAZ3/DAZ4 pair, which involved the region extending at least from position 978 to position 2071 in Fragment II. Each one of the above-mentioned three variations was otherwise observed in the control panel.

## Discussion

### Keystone of the novel approach–Determination of the variant ratio at a series of SFV positions

Both aspects of this study, namely determining the AZFc partial deletion/duplication status and discriminating the various combinations of deleted or duplicated DAZ family members, are based on the knowledge of the variant ratio at a series of SFV positions. Variant ratios are established by semi-quantitative sequencing, a method that has successfully been used for determination of allele frequency in pooled DNA samples [32] as well as relative quantification of virus population size in mixed genotype infections, among others [33].

### Suitability of variant ratio analysis to identify partial deletion and partial duplication samples

Apart from STS analysis, the most frequently used methods for the identification of AZFc partial deletion and duplication include real-time quantitative PCR [18,34,35], FISH [36], dosage PCR [5,15,17,31,37] and dosage Southern blot [12,37] by determining the cumulative copy number of the DAZ and other gene families. FISH is thought to be reliable but quite laborious, as it requires the use of whole cells. In addition to being labor-intensive, too, dosage Southern blot can be impaired by either gene conversion or the presence of an unexpected SNP at any restriction site. Real-time or dosage PCR evaluation, at the same time, may be biased by either imbalanced amplification or intra-locus segmental copy number variations affecting the region chosen to be amplified. Concordant results of two quantitative PCR assays designed to distant regions of the DAZ genes may be necessary for the secure determination of the family’s cumulative copy number [35]. Since variant ratio analysis utilizes the horizontal variant ratio distribution in order to determine the DAZ gene family’s partial deletion or duplication status, conclusions are based on a series of data instead of one density/intensity or C_t_ reading. The requirement for high concordance between the findings derived from two distantly located long (>2 kb) fragments is expected to offer protection against the bias caused by the above-mentioned factors. All deletions identified by variant ratio analysis were confirmed by the lack of certain STS markers as well as the results of a widely accepted DAZ dosage test. The latter also confirmed all duplications found on the basis of the horizontal variant ratio distribution.

Grouping samples by the horizontal variant ratio distribution is based on partial rearrangement-induced changes in the ratio of the copy number of the DAZ family members, while dosage tests directly measure the cumulative copy number of the gene family. Both methods have some shortcomings with respect to revealing certain AZFc rearrangements. The horizontal variant ratio distribution is unable to distinguish partial deletions from partial deletions followed by duplication, and it is also insensitive to identify samples with the entire AZFc region duplicated. At the same time, dosage tests cannot make distinction between unaffected samples and those that underwent partial deletion followed by duplication. For these reasons, variant ratio analysis is recommended to be complemented by a dosage test. The integration of the two methods makes it possible to differentiate all the above-mentioned structures. However, dividing samples into partial deletion, control and partial duplication groups for the purpose of subtyping should be made on the basis of the horizontal variant ratio distribution. Samples with partial deletion followed by duplication have four DAZ copies but they must undergo subtyping. At the same time, samples with the entire AZFc region duplicated possess eight DAZ genes but the 1:1:1:1 ratio of the four DAZ family members makes them eligible to be included in the control group. Nevertheless, based on the results of the dosage analysis, neither of these two rearrangement types was identified among our samples.

Although it is not typical, the results of several studies suggest the existence of deletions eliminating only one DAZ gene [3,38]. While the potential mechanisms that may result in such deletions are unknown, the possibility of their occurrence can’t be excluded. In spite of the fact that we have not found any sample containing three DAZ genes, the method described in this paper is thought to be able to discriminate them from samples possessing two, four or six DAZ copies. As opposed to the horizontal variant ratio distribution, a dosage test is not expected to reliably discriminate three copies of a target region from four.

### Determination of the concrete applicability of family member-specific variants as markers

To distinguish the members of a gene family from one another would normally require the use of sequence elements that (*i*) are characteristic of the respective members and (*ii*) do not show inter-individual variation. Several studies utilized genetic traits supposed to fulfill the above requirements for the discrimination of the various DAZ deletion or duplication subtypes [3–5,12,38]. However, little information has been reported on the inter-individual variation regarding either the actual marker-carrying DAZ family members [see requirement c (S6 Table)] or the copy number [0, 1 or 2 (S8 File)] of the tested “family member-specific” features in a control population consisting of individuals who carry one copy of each member of the DAZ gene family [14]. Based on the ability of variant ratio analysis to sort out samples according to partial rearrangement status, we could easily construct a control panel suitable to tackle this task before subtyping partial deletion and partial duplication samples. The inter-individual variability found at the majority of the studied SFV positions among our controls makes the importance of weighting marker copy number data obvious in order to draw valid conclusions for the copy number of gene family members.

The analysis to identify the different DAZ deletion and duplication subtypes utilizes the knowledge of the copy number of the DAZ family member-specific variant(s) at several SFV positions as a starting point (S8 File). Candidate markers designated on the basis of the reference sequence are divided into four classes according to observations made in a set of control samples. The applicability of markers is then determined for each class severally. The restricted applicability is derived by the analysis of the separate markers (S1 and S2 Files), while the extended applicability is determined on the basis of their observed associations (S3 and S4 Files). Ideally, such a procedure would require the knowledge of the spectrum of actually operating gene conversions resulting in the deletion of a class II/b and/or the duplication of a class II/a marker within a population or haplogroup. In the lack of that knowledge, we can specify that spectrum on the basis of the observations made in the control group.

Stage 3 analysis uses the extended applicability for markers that take part in an association and the restricted applicability for all others. During the analysis of our samples, as opposed to a kind of traditional (i.e. stage 1) evaluation, stage 3 analysis proved to be appropriate to assign a highly probable rearrangement subtype to all samples found partially deleted or duplicated by the horizontal variant ratio distribution (S8 and S9 Tables).

### Looking for perfect concordance–Possible ways to support the correctness of the assigned subtypes

The concordance can be determined per sample for each potential rearrangement subtype. At stage 3, it is 100% for the assigned subtype in almost all cases. The only exception is Ydel_07 with 92.86%. The concordance for any other subtype is typically lower than 30% in all deletion and duplication samples, but never exceeds 67%. Based on the concordance, it is possible to determine the likelihood of each rearrangement subtype in a sample. In the majority of samples, the likelihood of the assigned subtype is higher than 0.5, while that of the second most probable subtype is typically lower than 0.2. That substantial difference could be achieved due to the usage of the revised applicability of the studied markers.

The comparison of the VRH of a partial deletion or partial duplication sample with those that can theoretically be derived by the concluded type of rearrangement from the VRHs identified in the control panel is a further way to support the pertinence of the conclusion. A picturesque example for this is the case of samples Ydel_09 and Ydel_10. Their DAZ2/DAZ4 deletion subtype seemed to be unambiguous at stage 1 analysis. However, due to the non-informative nature of both the presence of the class II/a DAZ3-specific cluster and the absence of the class II/b DAZ4-specific markers, only the presence of DAZ1 and the absence of DAZ2 remained certain at stage 2. After that, these samples can theoretically have either DAZ2/DAZ4 or DAZ2/DAZ3 deletion. According to the association between A_972_ and C_1820_, it could be supposed that the DAZ4-specific C_1820_ was removed by something other than gene conversion. If DAZ4 is deleted, the DAZ3-specific cluster must be on DAZ3, in accordance with the association between the disappearance of the DAZ4-specific and the doubling of the DAZ3-specific markers on Fragment II. That is to say, the assigned deletion subtype at stage 3 was DAZ2/DAZ4 again. The VRH comparison indicates that there are two control VRHs whose rearrangement by DAZ2/DAZ4 deletion would result in the same series of variant ratios as that found in samples Ydel_09 and Ydel_10 (S11A Table). At the same time, there is no control VRH whose DAZ2/DAZ3 deletion would lead to the expected variant ratio series (S11B Table). These findings further confirm the propriety of DAZ2/DAZ4 deletion status concluded by stage 3 analysis for these samples.

In summary, the DAZ deletion/duplication subtype most consistent with the data provided by this method always seems to be the correct one.

### General considerations and possible extensions

The applicability of SFVs for the identification of deletions in the human Y chromosome has been challenged a decade ago [39]. It is obvious from previous efforts invested to make distinction among the various partial rearrangement types in AZFc that much caution is required to interpret the results obtained by the analysis of SFV positions. However, since we do not have other types of reliable markers in sufficient number, the information offered by SFV positions can be still very useful and is worthy trying to exploit to the best advantage. Several important aspects of the utilization of the information carried by SFVs must be emphasized here.

*First*, any improvement in the methodology that allows determining the variant ratio of an SFV position may add significantly to the success of subtyping AZFc partial rearrangements. RFLP has been the most frequently used method for AZFc subtyping [5,15,17]. In the view of a family member-specific variant, gene conversion can take place in two directions: it either eliminates or duplicates the variant. Restriction digestion can detect the loss of a family member-specific variant, but is insensitive to its duplication. This may have a bias on the evaluation of deletion samples. In addition, it makes RFLP insufficient for subtyping of duplication samples, since no variant loss is expected in them. Nevertheless, if made quantitative, this technique could also be used for variant ratio analysis. Despite this potentiality, semi-quantitative sequencing seems to be more suitable for the rapid and accurate determination of several variant ratios simultaneously. Massively parallel sequencing of PCR amplified fragments may make automation of variant ratio determination possible as well by taking advantage of simply counting the reads that contain different variants at an SFV position, instead of AUC measurements of fluorescent signals. The per-base coverage required to ensure specific variant reads frequencies with standard deviation low enough to reliably reflect the different variant ratios at a studied position may approximate or even exceed 1000-fold. However, the ever-increasing yield and decreasing costs of an average sequencing run made that high coverage easily attainable.

*Second*, in the absence of the knowledge of the common SFV organizations in the Y chromosome genealogical branches [39], characterization of the conversion profiles of the tested family member-specific SFVs by using a well-defined, extensive control panel seems obligatory [14]. We would like to underline that it must be done in each population studied, separately. The p1 and p2 values and the association pattern of the tested variants must preferably be re-evaluated after the examination of a new set of samples by summarizing all available data. Revealing the associations, which may be different from population to population, is of utmost importance for the success of subtyping. Alternatively, it is also possible to classify the studied variants in samples belonging to defined Y haplogroups. In that case, their haplogroup-restricted applicability could be used when analyzing haplogroup-typed samples. Some markers are expected to advance one class, or perhaps even two, when used within a given haplogroup. The larger the number of the higher class markers, the more unambiguous the outcome of the evaluation. Variant ratio analysis is suitable for the easy selection of control individuals carrying the four DAZ family members in a 1:1:1:1 ratio. To our knowledge, no method used so far for subtyping in AZFc region is suitable for marker classification.

*Third*, an important element of the presented method is the concordance of data obtained in the analysis of an individual. The more extensive is the concordance, the more likely the conclusion is correct. It is possible to expand the method by the inclusion of further SFV positions scattered along the entire length of the DAZ genes. We could identify three further long fragments, each containing several variants specific to different DAZ family members based on the reference sequence. The short amplicons that carry the well-known SNV sites (SNV I-VII) can also be involved into the analysis. Altogether, such a set of SFVs probably contains a number of variants large enough to ensure highly probable conclusions after marker classification and the establishment of the relationship between marker copy numbers and gene family member copy numbers. Since the SNVs have already been used in several populations, it can be supposed that the majority of the variants selected on the basis of the reference sequence are also eligible for analysis in samples which belong to Y haplogroups that are not close to the refseq. To test this hypothesis, we carried out a haplogroup analysis in our partial deletion and partial dulplication samples [40–41]. The results show that the 13 rearranged samples are split into four haplogroups or haplogroup clusters (S7 Table). The VRHs of the samples are in keeping with the identified haplogroups.

*Fourth*, the analysis can be extended to other gene families of AZFc to discriminate all combinations of deleted or duplicated family members [22]. Although the other seven AZFc gene families all have members with identical coding sequence, their regulation might differ. Most of them contain some intronic, upstream or downstream SFV positions that may make their differentiation possible. The rapid increase in the capacity of massively parallel sequencing has brought about the opportunity to evaluate several gene families in a large number of individuals with appropriate coverage in one sequencing run. By the preparation of a suitable evaluation script, the rearrangement subtypes can be obtained quickly and easily from next-generation sequencing (NGS) data. The ability to make clear distinction among the various combinations of deleted or duplicated family members will make it possible to conduct association studies to clarify their role in infertility and TGCTs. Another potential outcome of such studies can be the enrichment of certain VRHs within a deletion or duplication subtype, or even in unaffected samples, in the disease group compared to healthy individuals.

The novel approach described in this paper is thought to be able to eliminate some of the methodological bias that possibly affected the results of several subtyping studies carried out so far. By drawing an accurate picture of the studied SFV positions, the application of variant ratio analysis can be expected to contribute to the final settlement of the controversy regarding the role of this rearrangement-prone region in diseases.

This work is meant to be a proof-of-principle study that shows how it is possible to make distinction among the DAZ deletion and duplication subtypes with very high sensitivity by using a single, integrated approach. Based on the described principles, it is possible to develop an NGS-based method that would be easily applicable for rapid routine screening in clinical settings.

## Supporting Information

S1 Fig The base composition of the studied SFV positions in Fragments I and II. The base composition of the studied SFV positions as *expected* on the basis of the human reference assembly (hg18) (**upper part**). The cumulative length of the two amplified fragments is almost 5 kb. The four DAZ genes differ from one another at only several positions in these regions. The base residing in only one member at a given position is called the family member-specific variant. The base carried by the other three family members at the corresponding position is the non-specific variant. The ratio of the specific to the non-specific variant is expected to be 1:3 in unaffected samples. Supposing pairwise deletion and duplication, the ratio of the specific variant in deletion samples would be 1:1 (if two DAZ family members containing the non-specific variant are deleted) or 0:2 (if the member carrying the specific variant is deleted along with another one). A duplication event can modify the variant ratio to 1:5 (if two DAZ family members containing the non-specific variant are duplicated) or 2:4 (if the member carrying the specific variant is duplicated along with another one).
The base composition of the studied SFV positions as *found* on the basis of sequencing the members of the control panel (**lower part**). Contrasting the expectations, more than one typical electropherogram pictures were identified at the majority of the studied SFV positions. Only three positions (1209, 1702 and 1926, all in Fragment I) behaved identically in all 39 samples. The one letter codes of the relevant bases are shown above the electropherogram pictures. Variant ratios were assigned at each position on the basis of AUC ratio clusters in the knowledge of the AZFc partial deletion/duplication status and/or the AUC ratio–variant ratio relationship determined using appropriate control mixtures. Positions 1053, 1646 and 1961 in Fragment II are not shown due to lack of space.
(TIF)

S2 Fig Study of six STS markers using two multiplex PCR assays. sY1258, sY1201, sY1291, sY1191, sY1197 and sY1206: STS markers. Ydel_##: deletion sample identifier. Lane 1–4 and 6–8: deletion samples; lane 5 and 9–11: members of the control panel containing all four members of the DAZ gene family; lane 12: ladder; lane 13–16: control STS markers. The images indicate the existence of three different rearrangement types. Seven out of the eight deletion samples found in the experiment are included in the gel. None of the tested STS markers was missing in any control or duplication sample included in the study.
(TIF)

S1 File Relationship between the copy numbers of a class II/a DAZ3-specific marker and the copy numbers of the DAZ3 gene in deletion and duplication samples, respectively. (PDF)

S2 File Relationship between the copy numbers of a class II/b DAZ1-specific marker and the copy numbers of the DAZ1 gene in deletion and duplication samples, respectively. (PDF)

S3 File Relationship between the copy numbers of the associated class II/b DAZ1-specific A_972_ and class II/b DAZ4-specific C_1820_ markers and the copy numbers of the DAZ1 and DAZ4 genes in deletion and duplication samples, respectively. (PDF)

S4 File Relationship between the copy numbers of the associated class II/a DAZ3-specific and class II/b DAZ4-specific markers located in Fragment II and the copy numbers of the DAZ3 and DAZ4 genes in deletion and duplication samples, respectively. (PDF)

S5 File Alignment of the four amplicons constituting Fragment I. The sequences of the amplicons constituting Fragment I, which were derived from the hg18 reference sequence of the four DAZ genes, respectively, were aligned by Multalin sequence alignment tool. The Y chromosomal coordinates are seen at the beginning of the rows. DAZ1 and DAZ3 are designated by a “-”sign between the coordinates, while DAZ2 and DAZ4 with a “+” sign, according to the coding strand. The bases located in an SFV position are shown in blue. The family members from above are the following: DAZ1, DAZ2, DAZ3 and DAZ4.
(GIF)

S6 File Alignment of the four amplicons constituting Fragment II. The sequences of the amplicons constituting Fragment II, which were derived from the hg18 reference sequence of the four DAZ genes, respectively, were aligned by Multalin sequence alignment tool. The Y chromosomal coordinates are seen at the beginning of the rows. DAZ1 and DAZ3 are designated by a “-”sign between the coordinates, while DAZ2 and DAZ4 with a “+” sign, according to the coding strand. The bases located in an SFV position are shown in blue. The family members from above are the following: DAZ1, DAZ2, DAZ4 and DAZ3.
(GIF)

S7 File UCSC Custom Tracks design to visualize the SFVs utilized in this study. The Fragments track indicates the location of Fragments I and II in the four DAZ family members, respectively. The DAZ SFVs track shows the DAZ family member-specific and non-specific variants utilized in this study. Variants presented in red and blue are specific and non-specific for the DAZ family member displayed in the browser, respectively. For non-specific variants, a digit in square brackets indicates the DAZ family member for which the corresponding SFV position contains a specific variant. The often-used DAZ SNVs are also shown. Switching between DAZ family members is possible by entering the gene symbol in the Genome Browser window then clicking “go”.
(TXT)

S8 File Explanations for the difference between the restricted and extended applicability of markers and the advantages of stage 2 and 3 analysis over stage 1. (PDF)

S1 Table Attributes of the SFV positions covered by Fragment I based on the human reference genome NCBI36/hg18. (PDF)

S2 Table Attributes of the SFV positions covered by Fragment II based on the human reference genome NCBI36/hg18. (PDF)

S3 Table Primers used for the amplification and sequencing of Fragments I and II. (PDF)

S4 Table Composition of the control plasmid DNA mixtures. (PDF)

S5 Table Relationship between variant ratios and AUC ratios at two SFV positions in control DNA mixtures. (PDF)

S6 Table Association status of variants considered specific to the four DAZ family members, respectively, in four samples bearing three different VRHs. (PDF)

S7 Table Control variant ratio haplotypes (VRHs) supposed to underlie the identified rearrangements. (PDF)

S8 Table Comparison of stage 1, stage 2 and stage 3 analyses of partial deletion samples. (PDF)

S9 Table Comparison of stage 1, stage 2 and stage 3 analyses of partial duplication samples. (PDF)

S10 Table Variant ratios concordant with the possible rearrangement subtypes at SFV positions not utilized for stage 2 and 3 analyses. (PDF)

S11 Table Comparison of an actual deletion VRH with those theoretically produced by DAZ2/DAZ4 or DAZ2/DAZ3 deletions from all control VRHs, respectively. (PDF)
