# Correction: Discrimination of Deletion and Duplication Subtypes of the Deleted in Azoospermia Gene Family in the Context of Frequent Interloci Gene Conversion

**DOI:** 10.1371/journal.pone.0171396

**Published:** 2017-01-30

**Authors:** The PLOS ONE Staff

The following information is missing from the Funding section: 5) Spanish Ministry of Health (FIS grant PI14/01250) to CK.

Additionally, a portion of the caption for Table 1 is incorrectly displayed in the Results section. Please see the complete, correct Table 1 caption here. The publisher apologizes for the errors.

**Table 1:** Relationship between a sample’s AZFc partial deletion/duplication status and its horizontal variant ratio distribution.

AZFc partial deletion/duplication status	Type of SFV positions (#specific variant: #non-specific variant)						
	0:2x	2x:0	x:x	1:3	1:5	2:4	4:2
No partial rearrangement (x = 2)	+	-	+	+	-	-	-
Partial deletion affecting two DAZ family members (x = 1)	+	+	+	-	-	-	-
Partial deletion affecting two DAZ family members followed by duplication (x = 2)	+	+	+	-	-	-	-
Partial duplication affecting two DAZ family members (x = 3)	+	-	+	-	+	+	+

A + sign means that the corresponding type of SFV position may be present in a sample with the relevant deletion/duplication status. A – sign means that the corresponding type of SFV position is not expected in a sample with the relevant deletion/duplication status. The value of x is the function of the deletion/duplication status.

Supposing pairwise deletion and duplication of the DAZ family members, one of seven different variant ratios (0:2x, 2x:0, x:x, 1:3, 1:5, 2:4 and 4:2) can be assigned to an SFV position on the basis of its electropherogram picture. Except for 2x:0 and x:x, those ratios directly show the copy number of the family member-specific variant at their respective position. The horizontal variant ratio distribution means the distribution of the different types of SFV positions of a sample. The AZFc partial deletion/duplication status can be determined from the horizontal variant ratio distribution. The electropherogram picture of type 0:2x (0:2, 0:4 or 0:6), type 2x:0 (2:0 or 4:0) and type x:x (1:1, 2:2 or 3:3) sites appears identical, respectively. Their exact variant ratio and, in turn, the copy number of the specific variant at the 2x:0 and x:x type positions can be obtained from the AZFc partial deletion/duplication status of the sample. Subtyping uses both the AZFc partial deletion/duplication status and the copy number of the specific variant(s) at each SFV position as the starting point. [There are positions, such as position 1964 in Fragment II, which comprise more than two variants in certain samples; therefore, their description is necessarily more complex. For example, in the view of DAZ3, the integers in the formula 1:(1+2) mean one specific C, one non-specific A (which, at the same time, is specific to DAZ4) and two non-specific Gs. Overall, it refers to a 1:3 type position. Under the same considerations, 1:(0+3) is identical with 1:3; 0:(2+2) and 0:(1+3) with 0:4; and 2:(0+2) with 2:2.]

Based on variant ratios only, no distinction can be made between partial deletion and partial deletion followed by duplication. Therefore, samples found to carry partial deletion must be checked using a dosage test to determine if they also underwent duplication. In a similar way, the identification of samples with the entire AZFc region duplicated requires to subject partially-non-rearranged samples to a dosage test. However, the lack of knowledge of the exact DAZ copy number of samples belonging to these two categories does not influence subtyping.
