# Supplementary figures and images for: Discrimination of Deletion and Duplication Subtypes of the Deleted in Azoospermia Gene Family in the Context of Frequent Interloci Gene Conversion
Source: PLoS One. 2016 Oct 10;11(10):e0163936. doi: 10.1371/journal.pone.0163936 (PMC5056753; doi:10.1371/journal.pone.0163936)

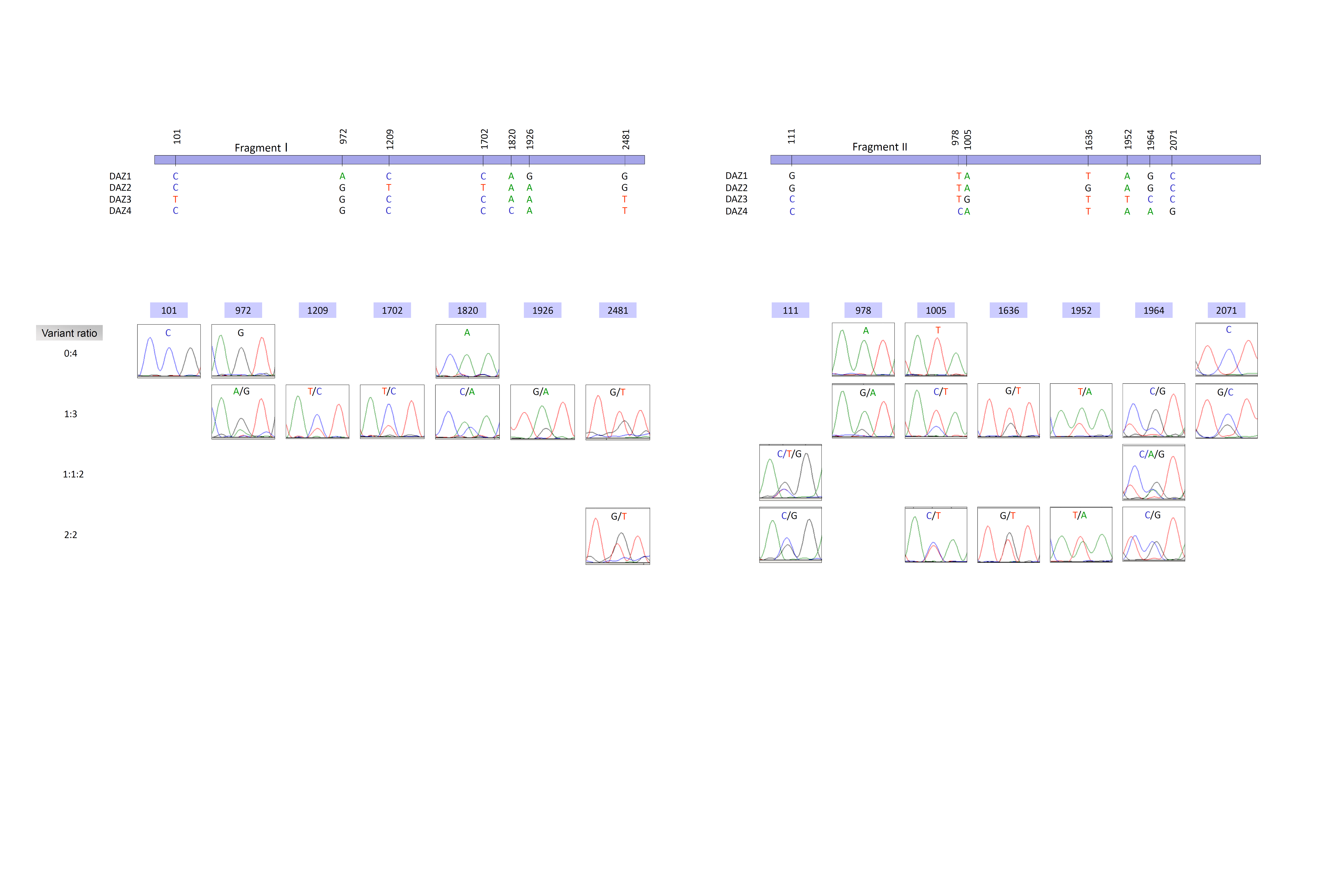

Supplement: S1 Fig — The base composition of the studied SFV positions as expected on the basis of the human reference assembly (hg18) (upper part). The cumulative length of the two amplified fragments is almost 5 kb. The four DAZ genes differ from one another at only several positions in these regions. The base residing in only one member at a given position is called the family member-specific variant. The base carried by the other three family members at the corresponding position is the non-specific variant. The ratio of the specific to the non-specific variant is expected to be 1:3 in unaffected samples. Supposing pairwise deletion and duplication, the ratio of the specific variant in deletion samples would be 1:1 (if two DAZ family members containing the non-specific variant are deleted) or 0:2 (if the member carrying the specific variant is deleted along with another one). A duplication event can modify the variant ratio to 1:5 (if two DAZ family members containing the non-specific variant are duplicated) or 2:4 (if the member carrying the specific variant is duplicated along with another one). The base composition of the studied SFV positions as found on the basis of sequencing the members of the control panel (lower part). Contrasting the expectations, more than one typical electropherogram pictures were identified at the majority of the studied SFV positions. Only three positions (1209, 1702 and 1926, all in Fragment I) behaved identically in all 39 samples. The one letter codes of the relevant bases are shown above the electropherogram pictures. Variant ratios were assigned at each position on the basis of AUC ratio clusters in the knowledge of the AZFc partial deletion/duplication status and/or the AUC ratio–variant ratio relationship determined using appropriate control mixtures. Positions 1053, 1646 and 1961 in Fragment II are not shown due to lack of space. (TIF) [file pone.0163936.s001.tif]

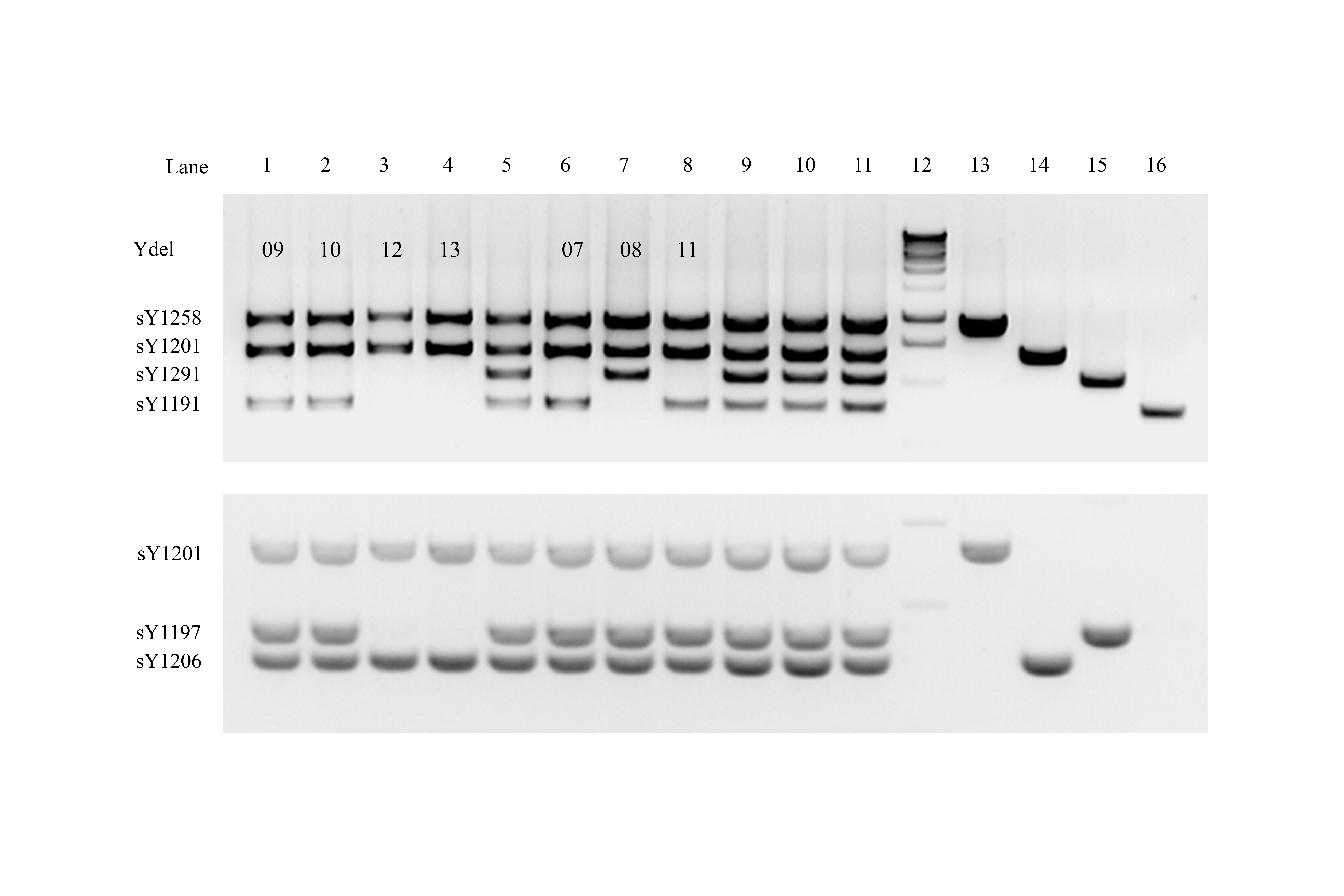

Supplement: S2 Fig — sY1258, sY1201, sY1291, sY1191, sY1197 and sY1206: STS markers. Ydel_##: deletion sample identifier. Lane 1–4 and 6–8: deletion samples; lane 5 and 9–11: members of the control panel containing all four members of the DAZ gene family; lane 12: ladder; lane 13–16: control STS markers. The images indicate the existence of three different rearrangement types. Seven out of the eight deletion samples found in the experiment are included in the gel. None of the tested STS markers was missing in any control or duplication sample included in the study. (TIF) [file pone.0163936.s002.tif]

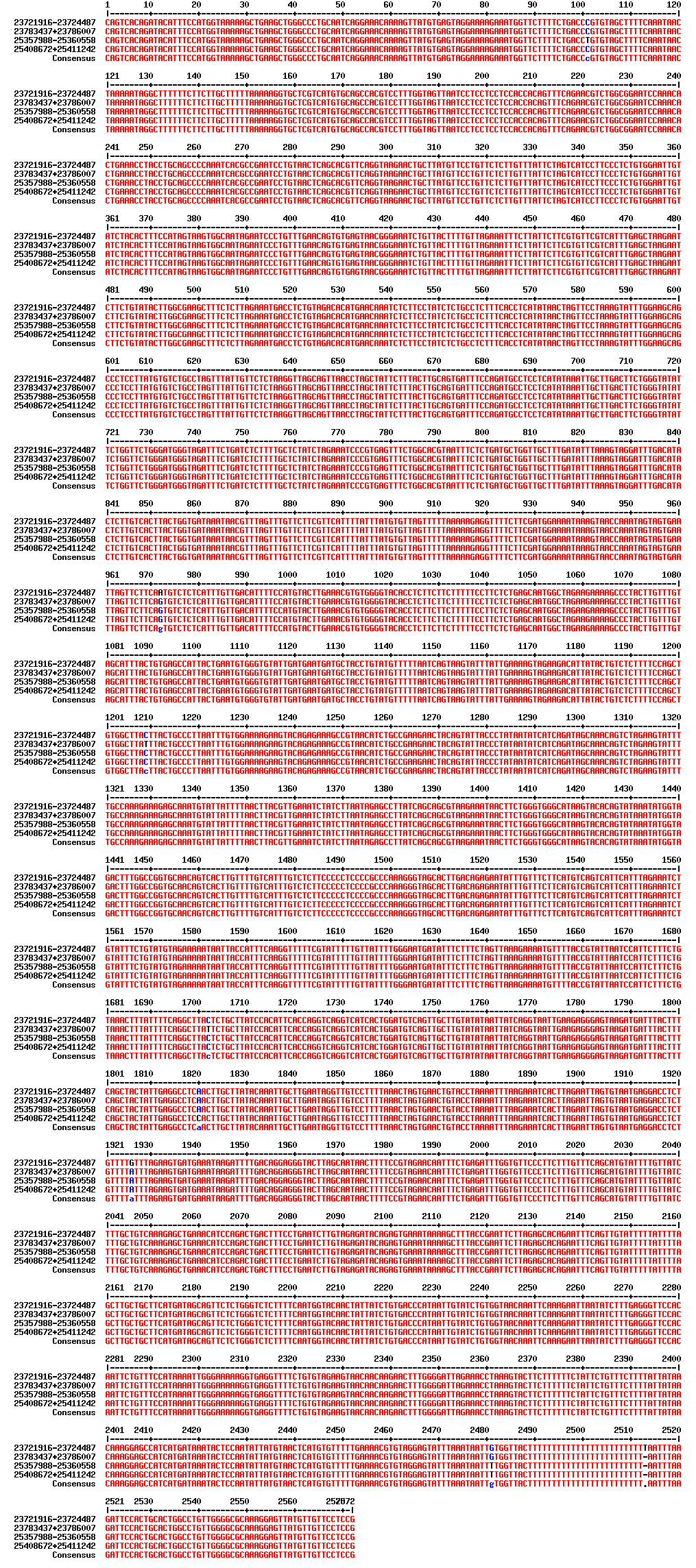

Supplement: S5 File — The sequences of the amplicons constituting Fragment I, which were derived from the hg18 reference sequence of the four DAZ genes, respectively, were aligned by Multalin sequence alignment tool. The Y chromosomal coordinates are seen at the beginning of the rows. DAZ1 and DAZ3 are designated by a “-”sign between the coordinates, while DAZ2 and DAZ4 with a “+” sign, according to the coding strand. The bases located in an SFV position are shown in blue. The family members from above are the following: DAZ1, DAZ2, DAZ3 and DAZ4. (GIF) [file pone.0163936.s007.gif]

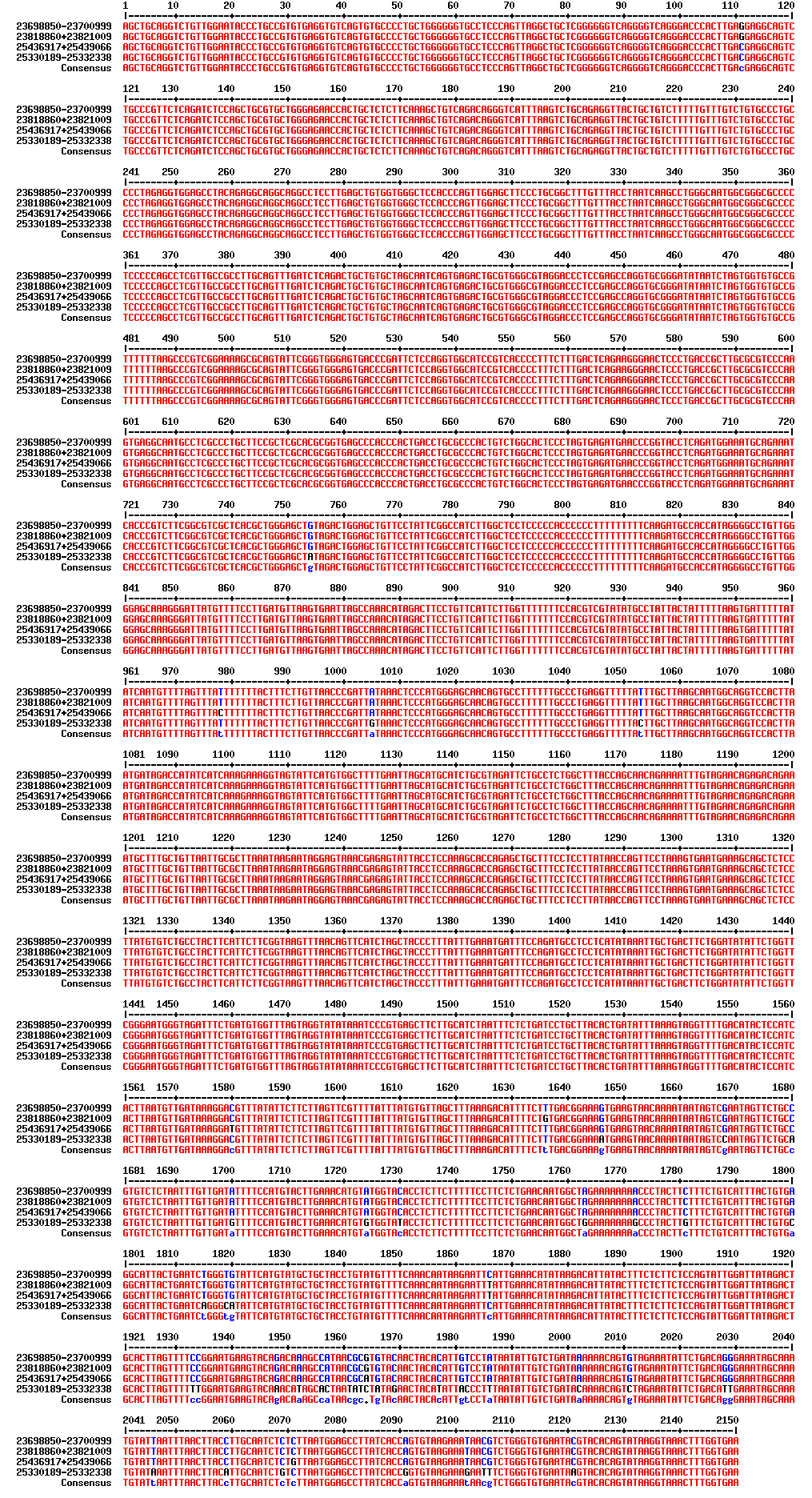

Supplement: S6 File — The sequences of the amplicons constituting Fragment II, which were derived from the hg18 reference sequence of the four DAZ genes, respectively, were aligned by Multalin sequence alignment tool. The Y chromosomal coordinates are seen at the beginning of the rows. DAZ1 and DAZ3 are designated by a “-”sign between the coordinates, while DAZ2 and DAZ4 with a “+” sign, according to the coding strand. The bases located in an SFV position are shown in blue. The family members from above are the following: DAZ1, DAZ2, DAZ4 and DAZ3. (GIF) [file pone.0163936.s008.gif]
