# Supplementary material for: Discrimination of Deletion and Duplication Subtypes of the Deleted in Azoospermia Gene Family in the Context of Frequent Interloci Gene Conversion
Source: PLoS One. 2016 Oct 10;11(10):e0163936. doi: 10.1371/journal.pone.0163936 (PMC5056753; doi:10.1371/journal.pone.0163936)
Supplement: S1 File — (PDF) [file pone.0163936.s003.pdf]

Supporting File S1. Relationship between the copy numbers of a class II/a DAZ3-specific marker and the copy numbers of the DAZ3 family member in deletion and duplication samples, respectively

Deletion	Copy number of DAZ3	Copy number of DAZ3-specific marker						
		No conversion	Gene conversion before deletion			Gene conversion after deletion		
			DAZ3>DAZ4	DAZ3>DAZ2	DAZ3>DAZ1	DAZ3>DAZ4	DAZ3>DAZ2	DAZ3>DAZ1
DAZ1/DAZ2	1	1	2	1	1	2	1	1
DAZ1/DAZ3	0	0	1	1	0	0	0	0
DAZ2/DAZ4	1	1	1	1	2	1	1	2
DAZ3/DAZ4	0	0	0	1	1	0	0	0
DAZ2/DAZ3	0	0	1	0	1	0	0	0
DAZ1/DAZ4	1	1	1	2	1	1	2	1

Duplication	Copy number of DAZ3	Copy number of DAZ3-specific marker						
		No conversion	Gene conversion before duplication			Gene conversion after duplication		
			DAZ3>DAZ4	DAZ3>DAZ2	DAZ3>DAZ1	DAZ3>DAZ4	DAZ3>DAZ2	DAZ3>DAZ1
DAZ1/DAZ2	1	1	2	3	3	2	2	2
DAZ1/DAZ3	2	2	3	3	4	3	3	3
DAZ2/DAZ4	1	1	3	3	2	2	2	2
DAZ3/DAZ4	2	2	4	3	3	3	3	3
DAZ2/DAZ3	2	2	3	4	3	3	3	3
DAZ1/DAZ4	1	1	3	2	3	2	2	2

The copy number of DAZ3 can only be changed by large rearrangements. At the same time, both large rearrangements and gene conversions may result in changes in the copy number of a DAZ3-specific marker.
Theoretically, DAZ3 may transfer the specific variant to any other DAZ family member by gene conversion while DAZ3 itself remains unchanged. Consequently, the DAZ3-specific variant's copy number doubles. Gene conversion can occur either before or after a large rearrangement event.
Only copy numbers of a specific variant unambiguously indicating the copy number of the relevant DAZ family member may be used for subtyping. The applicable copy numbers are emphasized by colored background.

The applicable marker copy numbers for deletion samples are the following:
Marker copy number 0 indicates gene family member copy number 0 (green).
Marker copy number 2 indicates gene family member copy number 1 (blue).

The applicable marker copy numbers for duplication samples are the following:
Marker copy number 1 indicates gene family member copy number 1 (green).
Marker copy number 4 indicates gene family member copy number 2 (blue).

The situation is the same with class II/a variants specific to other DAZ family members.
