# Supplementary material for: Discrimination of Deletion and Duplication Subtypes of the Deleted in Azoospermia Gene Family in the Context of Frequent Interloci Gene Conversion
Source: PLoS One. 2016 Oct 10;11(10):e0163936. doi: 10.1371/journal.pone.0163936 (PMC5056753; doi:10.1371/journal.pone.0163936)
Supplement: S2 File — (PDF) [file pone.0163936.s004.pdf]

Supporting File S2. Relationship between the copy numbers of a class II/b DAZ1-specific marker and the copy numbers of the DAZ1 family member in deletion and duplication samples, respectively

Deletion	Copy number of DAZ1	Copy number of DAZ1-specific marker						
		No conversion	Gene conversion before deletion			Gene conversion after deletion		
			DAZ2>DAZ1	DAZ4>DAZ1	DAZ3>DAZ1	DAZ2>DAZ1	DAZ4>DAZ1	DAZ3>DAZ1
DAZ1/DAZ2	0	0	0	0	0	0	0	0
DAZ1/DAZ3	0	0	0	0	0	0	0	0
DAZ2/DAZ4	1	1	0	0	0	1	1	0
DAZ3/DAZ4	1	1	0	0	0	0	1	1
DAZ2/DAZ3	1	1	0	0	0	1	0	1
DAZ1/DAZ4	0	0	0	0	0	0	0	0

Duplication	Copy number of DAZ1	Copy number of DAZ1-specific marker						
		No conversion	Gene conversion before duplication			Gene conversion after duplication		
			DAZ2>DAZ1	DAZ4>DAZ1	DAZ3>DAZ1	DAZ2>DAZ1	DAZ4>DAZ1	DAZ3>DAZ1
DAZ1/DAZ2	2	2	0	0	0	1	1	1
DAZ1/DAZ3	2	2	0	0	0	1	1	1
DAZ2/DAZ4	1	1	0	0	0	0	0	0
DAZ3/DAZ4	1	1	0	0	0	0	0	0
DAZ2/DAZ3	1	1	0	0	0	0	0	0
DAZ1/DAZ4	2	2	0	0	0	1	1	1

The copy number of DAZ1 can only be changed by large rearrangements. At the same time, both large rearrangements and gene conversions may result in changes in the copy number of a DAZ1-specific marker.
Theoretically, any of DAZ2, DAZ3 and DAZ4 may eliminate a class II/b DAZ1-specific variant by gene conversion while the donor family member remains unchanged. Consequently, the DAZ1-specific variant disappears at the relevant SFV position. Gene conversion can occur either before or after a large rearrangement event.
Only copy numbers of a specific variant unambiguously indicating the copy number of the relevant DAZ family member may be used for subtyping. The applicable copy numbers are emphasized by colored background.

The single applicable marker copy number for deletion samples is the following:
Marker copy number 1 indicates family member copy number 1 (green).

The single applicable marker copy number for duplication samples is the following:
Marker copy number 2 indicates gene family member copy number 2 (green).

The situation is the same with class II/b variants specific to other DAZ family members.
