# Supplementary material for: Discrimination of Deletion and Duplication Subtypes of the Deleted in Azoospermia Gene Family in the Context of Frequent Interloci Gene Conversion
Source: PLoS One. 2016 Oct 10;11(10):e0163936. doi: 10.1371/journal.pone.0163936 (PMC5056753; doi:10.1371/journal.pone.0163936)
Supplement: S3 File — (PDF) [file pone.0163936.s005.pdf]

Supporting File S3. Relationship between the copy numbers of the associated class II/b DAZ1-specific A_{972} and class II/b DAZ4-specific C_{1820} markers and the copy numbers of the DAZ1 and DAZ4 family members in deletion and duplication samples, respectively

Deletion	Copy number of DAZ family members		Copy number of DAZ1-specific A_{972} and DAZ4-specific C_{1820} markers in Fragment I													
			No conversion		DAZ1>DAZ4 and DAZ4>DAZ1 combined conversion				DAZ3>DAZ4 and DAZ2>DAZ1 combined conversion				DAZ2>DAZ4 and DAZ3>DAZ1 combined conversion			
					Conversion before deletion		Conversion after deletion		Conversion before deletion		Conversion after deletion		Conversion before deletion		Conversion after deletion	
	DAZ1	DAZ4	DAZ1_A_{972}	DAZ4_C_{1820}	DAZ1_A_{972}	DAZ4_C_{1820}	DAZ1_A_{972}	DAZ4_C_{1820}	DAZ1_A_{972}	DAZ4_C_{1820}	DAZ1_A_{972}	DAZ4_C_{1820}	DAZ1_A_{972}	DAZ4_C_{1820}	DAZ1_A_{972}	DAZ4_C_{1820}
DAZ1/DAZ2	0	1	0	1	0	0	0	1	0	0	0	0	0	0	0	1
DAZ1/DAZ3	0	1	0	1	0	0	0	1	0	0	0	1	0	0	0	0
DAZ2/DAZ4	1	0	1	0	0	0	1	0	0	0	1	0	0	0	0	0
DAZ3/DAZ4	1	0	1	0	0	0	1	0	0	0	0	0	0	0	1	0
DAZ2/DAZ3	1	1	1	1	0	0	0	0	0	0	1	1	0	0	1	1
DAZ1/DAZ4	0	0	0	0	0	0	0	0	0	0	0	0	0	0	0	0

Duplication	Copy number of DAZ family members		Copy number of DAZ1-specific A_{972} and DAZ4-specific C_{1820} markers in Fragment I													
			No conversion		DAZ1>DAZ4 and DAZ4>DAZ1 combined conversion				DAZ3>DAZ4 and DAZ2>DAZ1 combined conversion				DAZ2>DAZ4 and DAZ3>DAZ1 combined conversion			
					Conversion before duplication		Conversion after duplication		Conversion before duplication		Conversion after duplication		Conversion before duplication		Conversion after duplication	
	DAZ1	DAZ4	DAZ1_A_{972}	DAZ4_C_{1820}	DAZ1_A_{972}	DAZ4_C_{1820}	DAZ1_A_{972}	DAZ4_C_{1820}	DAZ1_A_{972}	DAZ4_C_{1820}	DAZ1_A_{972}	DAZ4_C_{1820}	DAZ1_A_{972}	DAZ4_C_{1820}	DAZ1_A_{972}	DAZ4_C_{1820}
DAZ1/DAZ2	2	1	2	1	0	0	1	0	0	0	1	0	0	0	1	0
DAZ1/DAZ3	2	1	2	1	0	0	1	0	0	0	1	0	0	0	1	0
DAZ2/DAZ4	1	2	1	2	0	0	0	1	0	0	0	1	0	0	0	1
DAZ3/DAZ4	1	2	1	2	0	0	0	1	0	0	0	1	0	0	0	1
DAZ2/DAZ3	1	1	1	1	0	0	0	0	0	0	0	0	0	0	0	0
DAZ1/DAZ4	2	2	2	2	0	0	1	1	0	0	1	1	0	0	1	1

Three combined gene conversions are supposed to be able to simultaneously eliminate the DAZ1-specific A_{972} and the DAZ4-specific C_{1820}. The gene conversions can occur either before or after a large rearrangement event.
Only copy number pairs of the DAZ1-specific A_{972} and the DAZ4-specific C_{1820} unambiguously indicating the copy number of DAZ1 and DAZ4 may be used for subtyping. The applicable pairs are emphasized by colored background.
For example, one copy of DAZ1_A_{972} and zero copies of DAZ4_C_{1820} refers to one copy of DAZ1 and zero copies of DAZ4 in deletion samples and two copies of DAZ1 and one copy of DAZ4 in duplication samples, respectively.

The applicable marker copy number pairs for deletion samples are the following:
The pair of DAZ1_A_{972} copy number 0 and DAZ4_C_{1820} copy number 1 indicates DAZ1 copy number 0 and DAZ4 copy number 1 (green).
The pair of DAZ1_A_{972} copy number 1 and DAZ4_C_{1820} copy number 0 indicates DAZ1 copy number 1 and DAZ4 copy number 0 (blue).
The pair of DAZ1_A_{972} copy number 1 and DAZ4_C_{1820} copy number 1 indicates DAZ1 copy number 1 and DAZ4 copy number 1 (gold).

The applicable marker copy number pairs for duplication samples are the following:
The pair of DAZ1_A_{972} copy number 0 and DAZ4_C_{1820} copy number 1 indicates DAZ1 copy number 1 and DAZ4 copy number 2 (green).
The pair of DAZ1_A_{972} copy number 1 and DAZ4_C_{1820} copy number 2 indicates DAZ1 copy number 1 and DAZ4 copy number 2 (green).
The pair of DAZ1_A_{972} copy number 1 and DAZ4_C_{1820} copy number 0 indicates DAZ1 copy number 2 and DAZ4 copy number 1 (blue).
The pair of DAZ1_A_{972} copy number 2 and DAZ4_C_{1820} copy number 1 indicates DAZ1 copy number 2 and DAZ4 copy number 1 (blue).
The pair of DAZ1_A_{972} copy number 2 and DAZ4_C_{1820} copy number 2 indicates DAZ1 copy number 2 and DAZ4 copy number 2 (gold).
The described relationship is based upon the observed association between the above markers and valid only for their copy numbers.
