# Supplementary material for: Discrimination of Deletion and Duplication Subtypes of the Deleted in Azoospermia Gene Family in the Context of Frequent Interloci Gene Conversion
Source: PLoS One. 2016 Oct 10;11(10):e0163936. doi: 10.1371/journal.pone.0163936 (PMC5056753; doi:10.1371/journal.pone.0163936)
Supplement: S4 File — (PDF) [file pone.0163936.s006.pdf]

Supporting File S4. Relationship between the copy numbers of the associated class II/a DAZ3-specific and class II/b DAZ4-specific markers and the copy numbers of the DAZ3 and DAZ4 family members in deletion and duplication samples, respectively

Deletion	Copy number of DAZ family members		Copy number of DAZ3- and DAZ4-specific markers located in Fragment II					
			No conversion		DAZ3>DAZ4 conversion			
					Conversion before deletion		Conversion after deletion	
	DAZ3	DAZ4	DAZ3_markers	DAZ4_markers	DAZ3_markers	DAZ4_markers	DAZ3_markers	DAZ4_markers
DAZ1/DAZ2	1	1	1	1	2	0	2	0
DAZ1/DAZ3	0	1	0	1	1	0	0	1
DAZ2/DAZ4	1	0	1	0	1	0	1	0
DAZ3/DAZ4	0	0	0	0	0	0	0	0
DAZ2/DAZ3	0	1	0	1	1	0	0	1
DAZ1/DAZ4	1	0	1	0	1	0	1	0

Duplication	Copy number of DAZ family members		Copy number of DAZ3- and DAZ4-specific markers located in Fragment II					
			No conversion		DAZ3>DAZ4 conversion			
					Conversion before duplication		Conversion after duplication	
	DAZ3	DAZ4	DAZ3_markers	DAZ4_markers	DAZ3_markers	DAZ4_markers	DAZ3_markers	DAZ4_markers
DAZ1/DAZ2	1	1	1	1	2	0	2	0
DAZ1/DAZ3	2	1	2	1	3	0	3	0
DAZ2/DAZ4	1	2	1	2	3	0	2	1
DAZ3/DAZ4	2	2	2	2	4	0	3	1
DAZ2/DAZ3	2	1	2	1	3	0	3	0
DAZ1/DAZ4	1	2	1	2	3	0	2	1

DAZ3>DAZ4 gene conversion is the most probable candidate to simultaneously double the copy number of DAZ3-specific markers and eliminate the DAZ4-specific markers, both located in Fragment II. The gene conversion can occur either before or after a large rearrangement event.
Only copy number pairs of a DAZ3- and a DAZ4-specific variant unambiguously indicating the copy number of DAZ3 and DAZ4 may be used for subtyping. The applicable pairs are emphasized by colored background.
For example, one copy of DAZ3- and one copy of DAZ4-specific markers as well as two copies of DAZ3- and zero copies of DAZ4-specific markers indicates the presence of one copy of DAZ3 and one copy of DAZ4 gene in both deletion and duplication samples.

The applicable marker copy number pairs for deletion samples are the following:
The pair of DAZ3-specific marker copy number 0 and DAZ4-specific marker copy number 0 indicates DAZ3 copy number 0 and DAZ4 copy number 0 (green).
The pair of DAZ3-specific marker copy number 0 and DAZ4-specific marker copy number 1 indicates DAZ3 copy number 0 and DAZ4 copy number 1 (blue).
The pair of DAZ3-specific marker copy number 1 and DAZ4-specific marker copy number 1 indicates DAZ3 copy number 1 and DAZ4 copy number 1 (gold).
The pair of DAZ3-specific marker copy number 2 and DAZ4-specific marker copy number 0 indicates DAZ3 copy number 1 and DAZ4 copy number 1 (gold).

The applicable marker copy number pairs for duplication samples are the following:
The pair of DAZ3-specific marker copy number 1 and DAZ4-specific marker copy number 1 indicates DAZ3 copy number 1 and DAZ4 copy number 1 (green).
The pair of DAZ3-specific marker copy number 2 and DAZ4-specific marker copy number 0 indicates DAZ3 copy number 1 and DAZ4 copy number 1 (green).
The pair of DAZ3-specific marker copy number 1 and DAZ4-specific marker copy number 2 indicates DAZ3 copy number 1 and DAZ4 copy number 2 (blue).
The pair of DAZ3-specific marker copy number 2 and DAZ4-specific marker copy number 2 indicates DAZ3 copy number 2 and DAZ4 copy number 2 (gold).
The pair of DAZ3-specific marker copy number 3 and DAZ4-specific marker copy number 1 indicates DAZ3 copy number 2 and DAZ4 copy number 2 (gold).
The pair of DAZ3-specific marker copy number 4 and DAZ4-specific marker copy number 0 indicates DAZ3 copy number 2 and DAZ4 copy number 2 (gold).
The described relationship is based upon the observed association between the above markers and valid only for their copy numbers.
