# Supplementary material for: Discrimination of Deletion and Duplication Subtypes of the Deleted in Azoospermia Gene Family in the Context of Frequent Interloci Gene Conversion
Source: PLoS One. 2016 Oct 10;11(10):e0163936. doi: 10.1371/journal.pone.0163936 (PMC5056753; doi:10.1371/journal.pone.0163936)
Supplement: S8 File — (PDF) [file pone.0163936.s010.pdf]

Supporting File S8. Explanations for the difference between the restricted and extended applicability of markers and the advantages of stage II and (if executable) stage III analysis over stage I

Conclusions from the copy numbers of a marker without marker classification													
**Usage: stage I analysis**	**Deletion sample**				**Control sample**				**Duplication sample**				
**Copy number of marker**	**0**	**1**	**2**		**0**	**1**	**2**		**0**	**1**	**2**	**3**	**4**
**Copy number of gene family member**	**0**	**1**	**?**		**1**	**1**	**1**		**0**	**1**	**2**	**?**	**?**

Based on our observations, the copy number of a gene family member-specific variant in a control sample, where the copy number of the the relevant gene family member is always 1, can be 0, 1 or 2 (Table 4). Without marker classification, nothing is known in advance about the actually occurring copy number(s) of a marker at a SFV position in a set of samples. Therefore, there is no chance to establish the relationship between the copy numbers of the marker and the copy numbers of the gene family member. There is nothing to do but accept the copy number of the marker as the copy number of the family member during the search for deletion and duplication samples and/or rearrangement subtypes.
The red letters indicate that the inference regarding the copy number of the relevant gene family member is uncertain.
The red question marks show that a gene family member copy number equivalent with the corresponding marker copy number is unexpected and unlikely.
An analysis of this kind is called stage I. We do not recommend to analyze samples in this way.

Conclusions from the copy numbers of markers after classifying them individually (restricted applicability)

Usage: stages II and III analyses	Deletion sample				Control sample				Duplication sample				
Copy number of class I marker	0	1				1				1	2		
Copy number of gene family member	0	1				1				1	2		
Copy number of class II/b marker	0	1			0	1			0	1	2		
Copy number of gene family member	-	1			1	1			-	-	2		
Copy number of class II/a marker	0	1	2			1	2			1	2	3	4
Copy number of gene family member	0	-	1			1	1			1	-	-	2
Copy number of class III marker	0	1	2		0	1	2		0	1	2	3	4
Copy number of gene family member	-	-	1		1	1	1		-	-	-	-	2

Marker classification is based on the copy numbers of a marker that are observed within the control group. It makes it possible to establish the relationship between the marker copy numbers and the the gene family member copy numbers in deletion and duplication samples, respectively.

As for class I markers, within the control group, their copy number is always identical with the copy number of the gene family member they are specific to. We suppose this relationship also holds true of deletion and duplication samples.

As far as class II/a and class II/b markers are concerned, their copy numbers vary within the control group. But the variation is limited to one direction (either elimination or duplication, but not both). Consequently, some marker copy numbers can inform about the copy number of the relevant gene family member, others not. For class III markers, copy number variation is observed in both directions. Their applicability is the union of that of class II/a and class II/b markers where non-informativity is dominant over informativity. Their applicability is very limited. However, they can be useful if there is no class II/a marker specific to the same gene family member.

Green fields show the copy numbers of the markers that unambiguously indicate the copy number of the relevant gene family member.

Red fields show the copy numbers of the markers that are non-informative about the copy number of the relevant gene family member.

The information indicated by the above table is obtained by the evaluation of the markers individually, not taking their potential associations into consideration.

This information is applied at stage II analysis for all markers, or, if there is (are) association(s) between some markers specific to different gene family members and thereby a stage III analysis can also be done, at stage III analysis for markers that do not participate in any association.

The way of establishing the relationship between the copy numbers of a marker and the copy numbers of the relevant gene family member can be seen in Supporting Files 1 and 2 for class II/a and II/b markers, respectively.

The restricted applicability for class II/a and II/b markers is shown by Tables 6 and 7, respectively.

Increment in the applicability of two class II/b markers specific to different gene family members due to their revealed association (restricted vs. extended applicability)

Conclusions from the copy numbers of two independent class II/b markers (restricted applicability)																		%
**Usage: stage II analysis**	**Deletion sample**				**Control sample**				**Duplication sample**									**38.46**
**Copy number of marker 1 (class II/b)**	0	0	1	1	0	0	1	1	0	0	0	1	1	1	2	2	2	**36.36**
**Copy number of marker 2 (class II/b)**	0	1	0	1	0	1	0	1	0	1	2	0	1	2	0	1	2	
**Copy number of gene family member 1**	-	-	1	1	1	1	1	1	-	-	-	-	-	-	2	2	2	
**Copy number of gene family member 2**	-	1	-	1	1	1	1	1	-	-	2	-	-	2	-	-	2	

The markers taking part in an association of this type in our samples are the DAZ1-specific A_{972} and the DAZ4-specific C_{1820}. If the two class II/b markers are not associated, 4 combinations of their copy numbers are expected in the control group. Independently of the marker copy number combinations, both gene family members are present in one copy in a control sample.

If the markers behave independently, 13 marker copy number combinations (4 in deletion samples and 9 in duplication samples) can theoretically exist. It means 26 data points (a marker copy number - gene family member copy number pair). Out of the 26, only 10 (38.46%) offer reliable copy number information about the relevant gene family member. Those data points are shown in green fields. The non-informative data points are indicated in red fields.

Conclusions from the copy numbers of two associated class II/b markers (extended applicability)																		%
**Usage: stage III analysis**	**Deletion sample**				**Control sample**				**Duplication sample**									**72.73**
**Copy number of marker 1 (class II/b)**	0	0	1	1	0			1	0	0	0	1	1	1	2	2	2	
**Copy number of marker 2 (class II/b)**	0	1	0	1	0			1	0	1	2	0	1	2	0	1	2	
**Copy number of gene family member 1**	-	0	1	1	1			1	-	1	NA	2	-	1	NA	2	2	
**Copy number of gene family member 2**	-	1	0	1	1			1	-	2	NA	1	-	2	NA	1	2	

According to our observations, the above two class II/b markers are associated. This inference came from the fact that only 2 combinations of their copy numbers could be found in the control group. Independently of the copy number combinations, both gene family members are present in one copy in a control sample.

Supposing the association of the markers, 11 marker copy number combinations (4 in deletion and 7 in duplication samples) can theoretically exist. It means 22 data points (a marker copy number - gene family member copy number pair). Out of the 22, as many as 16 (72.73%) offer reliable information. They are shown in green fields. The non-informative data points are indicated in red fields. Data points that refer to unexpected marker copy number combinations if the association exists are shown in yellow fields. A combination is unexpected if the difference in the copy number of the relevant markers is greater than 1. This rule comes from the nature of the association.

If there is an association, but the markers are considered as independent, only 8 out of the expected 22 data points (36.36%) can give information about the copy number of the relevant gene family member.

Summarizing the above, if there is an association between two class II/b markers specific to different gene family members, the information obtainable from the copy numbers of the relevant markers can be doubled by revealing the association.

The way of establishing the relationship between the copy numbers of an associated pair of two class II/b markers and the copy numbers of the relevant gene family members can be seen in Supporting File 3. The extended applicability of the two associated class II/b markers is shown by Table 8.

Increment in the applicability of a class II/a and a class II/b marker specific to different gene family members due to their revealed association (restricted vs. extended applicability)

Conclusions from the copy numbers of a class II/a and a class II/b marker which are independent from each other (restricted applicability)																							%
Usage: stage II analysis	Deletion sample						Control sample				Duplication sample												47.22
Copy number of marker 1 (class II/a)	0	0	1	1	2	2	1	1	2	2	1	1	1	2	2	2	3	3	3	4	4	4	38.46
Copy number of marker 2 (class II/b)	0	1	0	1	0	1	0	1	0	1	0	1	2	0	1	2	0	1	2	0	1	2	
Copy number of gene family member 1	0	0	-	-	1	1	1	1	1	1	1	1	1	-	-	-	-	-	-	2	2	2	
Copy number of gene family member 2	-	1	-	1	-	1	1	1	1	1	-	-	2	-	-	2	-	-	2	-	-	2	

The markers taking part in an association of this type in our samples are the class II/a DAZ3-specific and the class II/b DAZ4-specific markers in Fragment II. The association can actually be observed between the cluster of the relevant DAZ3- and the cluster of the relevant DAZ4-specific markers. If the above markers are not associated, 4 combinations of their copy numbers are expected in the control group. Independently of the marker copy number combinations, both gene family members are present in one copy in a control sample.
Using the markers independently, 18 marker copy number combinations (6 in deletion samples and 12 in duplication samples) can theoretically exist. It means 36 data points (a marker copy number - gene family member copy number pair). Out of the 36, only 17 (47.22%) are informative.

Conclusions from the copy numbers of an associated marker pair consisting of a class II/a and a class II/b marker (extended applicability)																							%
Usage: stage III analysis	Deletion sample						Control sample				Duplication sample												76.92
Copy number of marker 1 (class II/a)	0	0	1	1	2	2		1	2		1	1	1	2	2	2	3	3	3	4	4	4	
Copy number of marker 2 (class II/b)	0	1	0	1	0	1		1	0		0	1	2	0	1	2	0	1	2	0	1	2	
Copy number of gene family member 1	0	0	-	1	1	NA		1	1		NA	1	1	1	-	2	-	2	NA	2	NA	NA	
Copy number of gene family member 2	0	1	-	1	1	NA		1	1		NA	1	2	1	-	2	-	2	NA	2	NA	NA	

According to our observations, the above class II/a and class II/b markers are associated. This inference came from the fact that only 2 combinations of their copy numbers could be found in the control group. Independently of the copy number combinations, both gene family members are present in one copy in a control sample.
Supposing the association of the markers, 13 marker copy number combinations (5 in deletion samples and 8 in duplication samples) can theoretically exist. It means 26 data points (a marker copy number - gene family member copy number pair). Out of the 26, as many as 20 (76.92%) offer reliable gene copy number information.
The informative data points are shown in green fields. The non-informative ones are indicated in red fields. Data points referring to an unexpected scenario are shown in yellow fields. A marker copy number combination is unexpected if the sum of the copy number a DAZ3- and the copy number of a DAZ4-specific marker is greater than the maximum of the sum of the copy number of the relevant DAZ family members (2 in deletion and 4 in duplication samples). This rule comes from the nature of the association.
If there is an association, but it is not unveiled and the markers are considered to be independent, only 10 out of the expected 26 data points (38.46%) can give information about the copy number of the relevant gene family member.
Summarizing the above, if there is an association of this kind between a class II/a and a class II/b marker specific to different gene family members, the information theoretically obtainable from the copy numbers of the relevant markers can be doubled by revealing the association.
The way of establishing the relationship between the copy numbers of an associated pair of a class II/a and a class II/b marker and the copy numbers of the relevant gene family members can be seen in Supporting File 4. The extended applicability of the associated class II/a and class II/b markers is shown by Table 9.
