# Supplementary material for: Discrimination of Deletion and Duplication Subtypes of the Deleted in Azoospermia Gene Family in the Context of Frequent Interloci Gene Conversion
Source: PLoS One. 2016 Oct 10;11(10):e0163936. doi: 10.1371/journal.pone.0163936 (PMC5056753; doi:10.1371/journal.pone.0163936)
Supplement: S1 Table — (PDF) [file pone.0163936.s011.pdf]

Supporting Table S1. Attributes of the SFV positions covered by Fragment I as obtained from the human reference genome NCBI36/hg18.

Position in Fragment I		972	1209	1702	1820	1926	2481
Position in chrY	DAZ1	23723516	23723279	23722786	23722668	23722562	23722007
	DAZ2	23784408	23784645	23785138	23785256	23785362	23785917
	DAZ3	25359587	25359350	25358857	25358739	25358633	25358078
	DAZ4	25409643	25409880	25410373	25410491	25410597	25411152
Specific variant		A	T	T	C	G	-
Specificity		DAZ1	DAZ2	DAZ2	DAZ4	DAZ1	-
Non-specific variant(s)		G	C	C	A	A	G/T
Variant ratio		1A:3G	1T:3C	1T:3C	1C:3A	1G:3A	2G:2T[#]

[#] DAZ1/2: G, DAZ3/4: T
