# Supplementary material for: Discrimination of Deletion and Duplication Subtypes of the Deleted in Azoospermia Gene Family in the Context of Frequent Interloci Gene Conversion
Source: PLoS One. 2016 Oct 10;11(10):e0163936. doi: 10.1371/journal.pone.0163936 (PMC5056753; doi:10.1371/journal.pone.0163936)
Supplement: S2 Table — (PDF) [file pone.0163936.s012.pdf]

Supporting Table S2. Attributes of the SFV positions covered by Fragment II as obtained from the human reference genome NCBI36/hg18

Position in Fragment II		111	978	1005	1053	1636	1646	1952	1961	1964	1964	2071
Position in chrY	DAZ1	23700889	23700022	23699995	23699947	23699364	23699354	23699048	23699039	23699036	23699036	23698929
	DAZ2	23818970	23819837	23819864	23819912	23820495	23820505	23820811	23820820	23820823	23820823	23820930
	DAZ3	25332228	25331361	25331334	25331286	25330703	25330693	25330387	25330378	25330375	25330375	25330268
	DAZ4	25437027	25437894	25437921	25437969	25438552	25438562	25438868	25438877	25438880	25438880	25438987
Specific variant		-	C	G	C	G	A	T	T	C	A	G
Specificity		-	DAZ4	DAZ3	DAZ3	DAZ2	DAZ3	DAZ3	DAZ3	DAZ3	DAZ4	DAZ4
Non-specific variant(s)		G,C	T	A	T	T	G	A	C	G,A	G,C	C
Variant ratio		2G:2C#	1C:3T	1G:3A	1C:3T	1G:3T	1A:3G	1T:3A	1T:3C	1C:1A:2G	1A:1C:2G	1G:3C

#DAZ1/2: G, DAZ3/4: C
