# Supplementary material for: Discrimination of Deletion and Duplication Subtypes of the Deleted in Azoospermia Gene Family in the Context of Frequent Interloci Gene Conversion
Source: PLoS One. 2016 Oct 10;11(10):e0163936. doi: 10.1371/journal.pone.0163936 (PMC5056753; doi:10.1371/journal.pone.0163936)
Supplement: S3 Table — (PDF) [file pone.0163936.s013.pdf]

Supporting Table S3. Primers used for the amplification and sequencing of Fragments I and II

**Amplification primers**		
	Name	Sequence
Fragment I	MTY-I for	5′-CAG TCA CAG ATA CAT TTC CAT GGT A-3′
	MTY-I rev	5′-CGG AGG AAC AAC ATA ACT CCT T-3′
Fragment II	MTY-II for	5′-AGC TGC AGG TCT GTT GGA ATA-3′
	MTY-II rev	5′-TTC ACC AAA GTT TAC CTT ATA CTG TGT AC-3′
**Sequencing Primers**		
Fragment I	MTY-I for	5′-CAG TCA CAG ATA CAT TTC CAT GGT A-3′
	MTY-I_IntSeq-01 for	5′-CTG GTG ATA AAT AAC GTT TAG TTT GTT CT-3′
	MTY-I_IntSeq-02 for	5′-CCC AAA GGG TAG CAC TTG AC-3′
	MTY-I_IntSeq-03 for	5′-AAC AAA GGA GCC ATC ATG ATA AA-3′
Fragment II	MTY-II for	5′-AGC TGC AGG TCT GTT GGA ATA-3′
	MTY-II_IntSeq-01 rev	5′-GGT AAA GCC AGA GGC AGA AT-3′
	MTY-II_IntSeq-02 for	5′-CCG TGA GCT TCT TGC ATC TA-3′
	MTY-II rev	5′-TTC ACC AAA GTT TAC CTT ATA CTG TGT AC-3′
