# Supplementary material for: Discrimination of Deletion and Duplication Subtypes of the Deleted in Azoospermia Gene Family in the Context of Frequent Interloci Gene Conversion
Source: PLoS One. 2016 Oct 10;11(10):e0163936. doi: 10.1371/journal.pone.0163936 (PMC5056753; doi:10.1371/journal.pone.0163936)
Supplement: S4 Table — (PDF) [file pone.0163936.s014.pdf]

Supporting Table S4. Composition of the control plasmid DNA mixtures.

Mixture	Ratio of DAZ family member-specific insert-carrying plasmids				Rearrangement to mimic	Number of parallels sequenced
	DAZ1	DAZ2	DAZ3	DAZ4		
1	1	1	1	1	none	6
2	1	1	-	-	AZFc partial deletion eliminating DAZ3 and DAZ4	3
3	-	-	1	1	AZFc partial deletion eliminating DAZ1 and/DAZ2	3
4	1	-	1	-	AZFc partial deletion eliminating DAZ2 and DAZ4	3
5	-	1	-	1	AZFc partial deletion eliminating DAZ1 and DAZ3	3
6	2	2	1	1	AZFc partial duplication affecting DAZ1 and DAZ2	3
7	1	1	2	2	AZFc partial duplication affecting DAZ3 and DAZ4	3
8	2	1	2	1	AZFc partial duplication affecting DAZ1 and DAZ3	3
9	1	2	1	2	AZFc partial duplication affecting DAZ2 and DAZ4	3
