# Supplementary material for: Discrimination of Deletion and Duplication Subtypes of the Deleted in Azoospermia Gene Family in the Context of Frequent Interloci Gene Conversion
Source: PLoS One. 2016 Oct 10;11(10):e0163936. doi: 10.1371/journal.pone.0163936 (PMC5056753; doi:10.1371/journal.pone.0163936)
Supplement: S5 Table — (PDF) [file pone.0163936.s015.pdf]

Supporting Table S5. Relationship between variant ratios and AUC ratios at two SFV positions in control DNA mixtures

Position 1702 in Fragment I (DAZ2-specific)					
Variant ratio	0:2	1:5	1:3	2:4	1:1
AUC $ratio_{average}$	0.00	15.13	21.16	27.92	42.40
StDev	0.00	1.07	1.18	1.82	3.10
**Position 1926 in Fragment I (DAZ1-specific)**					
Variant ratio	0:2	1:5	1:3	2:4	1:1
AUC $ratio_{average}$	0.00	18.46	29.26	35.72	52.72
StDev	0.00	0.48	1.23	1.07	2.22

Each average and standard deviation value was calculated from six measurements.
