# Supplementary material for: Discrimination of Deletion and Duplication Subtypes of the Deleted in Azoospermia Gene Family in the Context of Frequent Interloci Gene Conversion
Source: PLoS One. 2016 Oct 10;11(10):e0163936. doi: 10.1371/journal.pone.0163936 (PMC5056753; doi:10.1371/journal.pone.0163936)
Supplement: S6 Table — (PDF) [file pone.0163936.s016.pdf]

Supporting Table S6a. Association status of variants considered specific to the four DAZ family members, respectively, in sample 4972 bearing VRH 2

Sample 4972, Fragment I							
SFV position	972	1209	1702	1820	1926	2481	
Specific variant	A	C	G	C	G	A	
DAZ specificity	DAZ1	DAZ2	DAZ2	DAZ4	DAZ1	-	
Variant ratios measured	1:3	1:3	1:3	1:3	1:3	2:2	Supposed origin
Consensus 1	A	C	C	A	G	G	DAZ1
Consensus 2	G	T	T	A	A	G	DAZ2
Consensus 3	G	C	C	A	A	T	DAZ3
Consensus 4	G	C	C	C	A	T	DAZ4

Sample 4972, Fragment II											
SFV position	111	978	1005	1053	1636	1646	1952	1961	1964	2071	
Specific variant	-	C	G	C	G	A	T	T	C	G	
DAZ specificity	-	DAZ4	DAZ3	DAZ3	DAZ2	DAZ3	DAZ3	DAZ3	DAZ3	DAZ4	
Variant ratios measured	2:2	1:3	1:3	1:3	1:3	1:3	1:3	1:3	1:3	1:3	Supposed origin
Consensus 1	G	T	A	T	T	G	A	C	G	C	DAZ1
Consensus 2	G	T	A	T	G	G	A	C	G	C	DAZ2
Consensus 3	C	T	G	C	T	A	T	T	C	C	DAZ3
Consensus 4	C	C	A	T	T	G	A	C	G	G	DAZ4

The association status is shown by the consensus variant series which were determined by cloning Fragments I and II and sequencing an appropriate number of colonies. The variant ratios determined by semi-quantitative sequencing as well as the DAZ family members supposed to correspond to the consensus variant series are also shown. Yellow, green, purple and blue rectangles indicate applicable DAZ1-, DAZ2-, DAZ3- and DAZ4-specific markers, respectively.

Supporting Table S6b. Association status of variants considered specific to the four DAZ family members, respectively, in sample 6079 bearing VRH 2

**Sample 6079, Fragment I**							
SFV position	972	1209	1702	1820	1926	2481	
Specific variant	A	C	G	C	G	A	
DAZ specificity	DAZ1	DAZ2	DAZ2	DAZ4	DAZ1	-	
Variant ratios measured	1:3	1:3	1:3	1:3	1:3	2:2	Supposed origin
Consensus 1	A	C	C	A	G	G	DAZ1
Consensus 2	G	T	T	A	A	G	DAZ2
Consensus 3	G	C	C	A	A	T	DAZ3
Consensus 4	G	C	C	C	A	T	DAZ4

**Sample 6079, Fragment II**											
SFV position	111	978	1005	1053	1636	1646	1952	1961	1964	2071	
Specific variant	-	C	G	C	G	A	T	T	C	G	
DAZ specificity	-	DAZ4	DAZ3	DAZ3	DAZ2	DAZ3	DAZ3	DAZ3	DAZ3	DAZ4	
Variant ratios measured	2:2	1:3	1:3	1:3	1:3	1:3	1:3	1:3	1:3	1:3	Supposed origin
Consensus 1	G	T	A	T	T	G	A	C	G	C	DAZ1
Consensus 2	G	T	A	T	G	G	A	C	G	C	DAZ2
Consensus 3	C	T	G	C	T	A	T	T	C	C	DAZ3
Consensus 4	C	C	A	T	T	G	A	C	G	G	DAZ4

The association status is shown by the consensus variant series which were determined by cloning Fragments I and II and sequencing an appropriate number of colonies. The variant ratios determined by semi-quantitative sequencing as well as the DAZ family members supposed to correspond to the consensus variant series are also shown. Yellow, green, purple and blue rectangles indicate applicable DAZ1-, DAZ2-, DAZ3- and DAZ4-specific markers, respectively.

Supporting Table S6c. Association status of variants considered specific to the four DAZ family members, respectively, in sample 5466 bearing VRH 3a/2

**Sample 5466, Fragment I**							
SFV position	972	1209	1702	1820	1926	2481	
Specific variant	A	C	G	C	G	A	
DAZ specificity	DAZ1	DAZ2	DAZ2	DAZ4	DAZ1	-	
Variant ratios measured	0:4	1:3	1:3	0:4	1:3	1:3	Supposed origin
Consensus 1	G	C	C	A	G	G	DAZ1
Consensus 2	G	T	T	A	A	T	DAZ2
Consensus 3	G	C	C	A	A	T	DAZ3
Consensus 3	G	C	C	A	A	T	DAZ4

**Sample 5466, Fragment II**											
SFV position	111	978	1005	1053	1636	1646	1952	1961	1964	2071	
Specific variant	-	C	G	C	G	A	T	T	C A	G	
DAZ specificity	-	DAZ4	DAZ3	DAZ3	DAZ2	DAZ3	DAZ3	DAZ3	3 4	DAZ4	
Variant ratios measured	1:1:2	1:3	1:3	1:3	2:2	1:3	1:3	1:3	1:1:2	1:3	Supposed origin
Consensus 1	G	T	A	T	G	G	A	C	G	C	DAZ1
Consensus 1	G	T	A	T	G	G	A	C	G	C	DAZ2
Consensus 2	T	T	G	C	T	A	T	T	C	C	DAZ3
Consensus 3	C	C	A	T	T	G	A	C	A	G	DAZ4

The association status is shown by the consensus variant series which were determined by cloning Fragments I and II and sequencing an appropriate number of colonies. The variant ratios determined by semi-quantitative sequencing as well as the DAZ family members supposed to correspond to the consensus variant series are also shown. Yellow, green, purple and blue rectangles indicate applicable DAZ1-, DAZ2-, DAZ3- and DAZ4-specific markers, respectively.

In the reference sequence, there is no specific variant at position 2481 in Fragment I (DAZ1/2:G, DAZ3/4:T). The results of this study raise the possibility that G_{2481} is a class II/a DAZ1-specific marker with p1=0.77 and p2=1.00. Its copy number is in accordance with the subtypes concluded for all deletion and duplication samples, respectively.

Supporting Table S6d. Association status of variants considered specific to the four DAZ family members, respectively, in sample 6100 bearing VRH 1

**Sample 6100, Fragment I**							
SFV position	972	1209	1702	1820	1926	2481	
Specific variant	A	C	G	C	G	A	
DAZ specificity	DAZ1	DAZ2	DAZ2	DAZ4	DAZ1	-	
Variant ratios measured	1:3	1:3	1:3	1:3	1:3	1:3	Supposed origin
Consensus 1	A	C	C	A	G	G	DAZ1
Consensus 2	G	T	T	A	A	T	DAZ2
Consensus 3	G	C	C	A	A	T	DAZ3
Consensus 4	G	C	C	C	A	T	DAZ4

**Sample 6100, Fragment II**											
SFV position	111	978	1005	1053	1636	1646	1952	1961	1964	2071	
Specific variant	-	C	G	C	G	A	T	T	C	G	
DAZ specificity	-	DAZ4	DAZ3	DAZ3	DAZ2	DAZ3	DAZ3	DAZ3	DAZ3	DAZ4	
Variant ratios measured	2:2	0:4	2:2	2:2	1:3	2:2	2:2	2:2	2:2	0:4	Supposed origin
Consensus 1	G	T	A	T	T	G	A	C	G	C	DAZ1
Consensus 2	G	T	A	T	G	G	A	C	G	C	DAZ2
Consensus 3	C	T	G	C	T	A	T	T	C	C	DAZ3
Consensus 3	C	T	G	C	T	A	T	T	C	C	DAZ4

The association status is shown by the four consensus variant series which were determined by cloning Fragments I and II and sequencing an appropriate number of colonies. The variant ratios determined by semi-quantitative sequencing as well as the DAZ family members supposed to correspond to the consensus series are also shown. Yellow, green, purple and blue rectangles indicate applicable DAZ1-, DAZ2-, DAZ3- and DAZ4-specific markers, respectively.

In the reference sequence, there is no specific variant at position 2481 in Fragment I (DAZ1/2:G, DAZ3/4:T). The results of this study raise the possibility that G_{2481} in Fragment I is class II/a DAZ1-specific marker with p1=0.77 and p2=1.00. Its copy number is in accordance with the subtypes concluded for all deletion and duplication samples, respectively.
