# Supplementary material for: Discrimination of Deletion and Duplication Subtypes of the Deleted in Azoospermia Gene Family in the Context of Frequent Interloci Gene Conversion
Source: PLoS One. 2016 Oct 10;11(10):e0163936. doi: 10.1371/journal.pone.0163936 (PMC5056753; doi:10.1371/journal.pone.0163936)
Supplement: S7 Table — (PDF) [file pone.0163936.s017.pdf]

Supporting Table S7. Control variant ratio haplotypes (VRHs) supposed to underlie the identified rearrangements

Sample identifier	Concluded rearrangement	VRH	Y haplogroups
Ydel_06*	DAZ3/4 deletion	3a/1#, 3b#	N
Ydel_08	DAZ3/4 deletion	3a/3	cluster CFGHI
Ydel_07*	DAZ3/4 deletion	2#	cluster PQR
Ydel_09	DAZ2/4 deletion	1; 2	cluster PQR
Ydel_10	DAZ2/4 deletion	1; 2	cluster PQR
Ydel_11	DAZ1/2 deletion	1	cluster PQR
Ydel_12	DAZ1/2 deletion	1	cluster PQR
Ydel_13	DAZ1/2 deletion	1	cluster PQR
Ydup_01*	DAZ3/4 duplication	3a/3#, 3b#	cluster CFGHI
Ydup_05	DAZ1/2 duplication	3b	cluster CFGHI
Ydup_03	DAZ2/4 duplication	1	cluster PQR
Ydup_04	DAZ2/4 duplication	1	cluster PQR
Ydup_02	DAZ3/4 duplication	3a/1	J

*No variant ratio haplotype was found among the controls which this sample could be directly derived from by the concluded type of rearrangement
#Control variant ratio haplotype most similar to that which the corresponding sample must have originated from by the concluded type of rearrangement

Analysis of Y haplogroups:
The human Y chromosomal haplogroups have been identified with a multi-step method described in [40] and adapted for the ABI Prism SNaPshot system [41]. This method analyzes simultaneously six binary markers in the non-recombinant portion of the human Y chromosome: M145, M9, M45, LLy22g, M96 and M168. The first step is a multiplex PCR that amplifies all binary markers. After an additional amplification, multiplexed primer-extension reactions are done with SNaPshot® Multiplex Kit (Thermo Fisher Scientific). The products are analyzed on an automatic sequencer (ABI PRISM 310 Genetic Analyzer PE). The detectable haplogroups or clusters of haplogroups are the following: A-B, DE, E, K-L-M-NO-O-S-T, N, P-Q-R, C-F-G-H-I and J. Given that C-F-G-H-I and J haplogroups show the same profile, a plus/minus PCR for the marker 12f2 was performed in order to distinguish between these two clusters.
