# Supplementary material for: Discrimination of Deletion and Duplication Subtypes of the Deleted in Azoospermia Gene Family in the Context of Frequent Interloci Gene Conversion
Source: PLoS One. 2016 Oct 10;11(10):e0163936. doi: 10.1371/journal.pone.0163936 (PMC5056753; doi:10.1371/journal.pone.0163936)
Supplement: S8 Table — (PDF) [file pone.0163936.s018.pdf]

Supporting Table S8a. Stage 1 analysis of deletion samples

Sample	DAZ1			DAZ2				DAZ3					DAZ4					Deleted
	Specific variants		Σ	Specific variants			Σ	Specific variants				Σ	Specific variants				Σ	
	I-A_{972}	I-G_{1926}		I-T_{1209}	I-T_{1702}	II-G_{1636}		II-A_{1646}	II-T_{1952}	II-T_{1961}	II-C_{1964}		I-C_{1820}	II-C_{978}	II-A_{1964}	II-G_{2071}		
Ydel_06	0	1	?	1	1	1	1	0	0	0	0	0	0	0	0	0	0	DAZ3/4 (?)
Ydel_08	0	1	?	1	1	1	1	0	0	0	0	0	0	0	0	0	0	DAZ3/4 (?)
Ydel_07	1	1	1	1	1	0	?	0	0	0	0	0	0	0	0	0	0	DAZ3/4 (?)
Ydel_09	1	1	1	0	0	0	0	1	1	1	1	1	0	0	0	0	0	DAZ2/4
Ydel_10	1	1	1	0	0	0	0	1	1	1	1	1	0	0	0	0	0	DAZ2/4
Ydel_11	0	0	0	0	0	0	0	2	2	2	2	2	1	0	0	0	?	DAZ1/2 (?)
Ydel_12	0	0	0	0	0	0	0	2	2	2	2	2	1	0	0	0	?	DAZ1/2 (?)
Ydel_13	0	0	0	0	0	0	0	2	2	2	2	2	1	0	0	0	?	DAZ1/2 (?)

The cells of „Specific variants" columns show the copy number of DAZ family member-specific variants determined by semi-quantitative sequencing at the respective SFV positions. The cells of „Σ" columns show the resultant conclusions concerning the copy number of the DAZ family members, which were derived by summarizing the individual data. It can be seen that the individual findings are contradictory for DAZ1, DAZ2 and DAZ4 in several samples. Moreover, in three samples, the copy number of DAZ3-specific markers is in complete accordance to indicate the presence of two copies of DAZ3, which is in conflict with the expectations based on the structure of AZFc region.

I-, II-: Fragment I and II
?: lack of conclusion due to controversy between findings
(?): uncertain since not all data are consistent with the concluded subtype

Supporting Table S8b. Stage 2 analysis of deletion samples

Sample	DAZ1			DAZ2				DAZ3					DAZ4					Deleted
	Specific variants		Σ	Specific variants			Σ	Specific variants				Σ	Specific variants				Σ	
	I-A_{972}	I-G_{1926}		I-T_{1209}	I-T_{1702}	II-G_{1636}		II-A_{1646}	II-T_{1952}	II-T_{1961}	II-C_{1964}		I-C_{1820}	II-C_{978}	II-A_{1964}	II-G_{2071}		
Ydel_06	x	1	1	1	1	x	1	0	0	0	0	0	x	x	x	x	?	DAZ3/4 (?)
Ydel_08	x	1	1	1	1	x	1	0	0	0	0	0	x	x	x	x	?	DAZ3/4 (?)
Ydel_07	1	1	1	1	1	0	?	0	0	0	0	0	x	x	x	x	?	DAZ3/4 (?)
Ydel_09	1	1	1	0	0	0	0	x	x	x	x	?	x	x	x	x	?	?
Ydel_10	1	1	1	0	0	0	0	x	x	x	x	?	x	x	x	x	?	?
Ydel_11	x	0	0	0	0	0	0	1	1	1	1	1	1	x	x	x	1	DAZ1/2
Ydel_12	x	0	0	0	0	0	0	1	1	1	1	1	1	x	x	x	1	DAZ1/2
Ydel_13	x	0	0	0	0	0	0	1	1	1	1	1	1	x	x	x	1	DAZ1/2

Based on the restricted applicability of markers determined by evaluating them *individually* after the classification procedure, findings considered unreliable indicators of the copy number of the respective DAZ family members could be excluded from the analysis.

The cells of „Specific variants" columns show the copy number of DAZ family members derived from the copy number of the specific variants according to the following:

(i) the copy number of a class I marker was considered to directly show the copy number of the relevant DAZ family member

(ii) class II/a and class II/b markers were evaluated according to the rules shown in Tables 6 and 7, respectively.

The cells of „Σ" columns show the resultant conclusions that were derived by summarizing the remaining individual data. The majority of contradictions have disappeared at this stage. The excess copy number of DAZ3 has also been resolved. However, in some cases, all data were lost for DAZ3 and DAZ4, which interfered with subtyping.

I-, II-: Fragment I and II
x: finding labeled non-informative and therefore excluded from the evaluation
?: lack of conclusion due to controversy between findings
?: lack of conclusion due to insufficient information
(?): uncertain due to insufficient information

Supporting Table S8c. Stage 3 analysis of deletion samples

Sample	DAZ1			DAZ2				DAZ3					DAZ4					Deleted
	Specific variants		Σ	Specific variants			Σ	Specific variants				Σ	Specific variants				Σ	
	I-A$_{972}$	I-G$_{1926}$		I-T$_{1209}$	I-T$_{1702}$	II-G$_{1636}$		II-A$_{1646}$	II-T$_{1952}$	II-T$_{1961}$	II-C$_{1964}$		I-C$_{1820}$	II-C$_{978}$	II-A$_{1964}$	II-G$_{2071}$		
Ydel_06	x	1	1	1	1	x	1	0	0	0	0	0	x	0	x	0	0	DAZ3/4
Ydel_08	x	1	1	1	1	x	1	0	0	0	0	0	x	0	x	0	0	DAZ3/4
Ydel_07	1	1	1	1	1	0	?	0	0	0	0	0	0	0	x	0	0	DAZ3/4
Ydel_09	1	1	1	0	0	0	0	x	x	x	x	?	0	x	x	x	0	DAZ2/4 (?)
Ydel_10	1	1	1	0	0	0	0	x	x	x	x	?	0	x	x	x	0	DAZ2/4 (?)
Ydel_11	0	0	0	0	0	0	0	1	1	1	1	1	1	1	x	1	1	DAZ1/2
Ydel_12	0	0	0	0	0	0	0	1	1	1	1	1	1	1	x	1	1	DAZ1/2
Ydel_13	0	0	0	0	0	0	0	1	1	1	1	1	1	1	x	1	1	DAZ1/2

The restricted applicability of several markers could be extended by *jointly* evaluating the members of marker pairs observed to be in association with each other. As a consequence, fewer data had to be excluded from the analysis then at stage 2.

The cells of „Specific variants" columns show the copy number of DAZ family members derived from the copy number of the specific variants according to the following:

(i) the copy number of a class I marker was considered to directly show the copy number of the relevant DAZ family member

(ii) class II/a and class II/b markers *showing no association* in the control panel (class II/a DAZ2-specific II-G$_{1636}$ and class II/b DAZ4-specific II-A$_{1964}$) were evaluated according to the rules displayed in Tables 6 and 7, respectively

(iii) markers *participating in one or the other of the two observed associations* were evaluated according to the rules shown in Tables 8 and 9, respectively.

The cells of „Σ" columns show the resultant conclusions for the copy number of the DAZ family members, which were derived by summarizing the individual findings. In addition to dissolving almost all contradictions, stage 3 analysis also provided enough data for reliable subtyping. In the case of Ydel_09 and Ydel_10, which could seemingly be subtyped with no doubt remaining at stage 1, there arose some uncertainty at stage 2, which could be resolved by deeper analysis (see in the text). The DAZ2/4 deletion status of these samples is also supported by VRH comparisons (Supporting Tables S10a-b).

I-, II-: Fragment I and II
x: finding labeled non-informative and therefore excluded from the evaluation
?: lack of conclusion due to controversy between findings
?: lack of conclusion due to insufficient information
