# Supplementary material for: Discrimination of Deletion and Duplication Subtypes of the Deleted in Azoospermia Gene Family in the Context of Frequent Interloci Gene Conversion
Source: PLoS One. 2016 Oct 10;11(10):e0163936. doi: 10.1371/journal.pone.0163936 (PMC5056753; doi:10.1371/journal.pone.0163936)
Supplement: S10 Table — (PDF) [file pone.0163936.s020.pdf]

Supporting table S10. Variant ratios concordant with the various rearrangement subtypes at positions not utilized for stage 2 and 3 analysis

	F-I 2481		F-II 111		F-II 1005			F-II 1053		
	G:T[1]		G:C:T[2]		G:A[3]			C:T[4]		
Starting variant ratio	2:2	1:3	2:2:0	2:1:1	1:3	2:2	0:4	1:3	2:2	0:4
DAZ1/2 deletion	0:2	0:2	0:2:0	0:1:1	1:1, 2:0	2:0	0:2	1:1, 2:0	2:0	0:2
DAZ3/4 deletion	2:0	1:1, 2:0	2:0:0	2:0:0	0:2	0:2	0:2	0:2	0:2	0:2
DAZ1/3 deletion	1:1	0:2	1:1:0	1:1:0	0:2	1:1	0:2	0:2	1:1	0:2
DAZ2/4 deletion	1:1	1:1	1:1:0	1:0:1	1:1	1:1	0:2	1:1	1:1	0:2
DAZ1/4 deletion	1:1	0:2	1:1:0	1:0:1	1:1	1:1	0:2	1:1	1:1	0:2
DAZ2/3 deletion	1:1	1:1	1:1:0	1:1:0	0:2	1:1	0:2	0:2	1:1	0:2
DAZ1/2 duplication	4:2	2:4, 1:5, 3:3	4:2:0	4:1:1	1:5, 0:6, 2:4	2:4	0:6	1:5, 0:6, 2:4	2:4	0:6
DAZ3/4 duplication	2:4	1:5, 0:6, 2:4	2:4:0	2:2:2	2:4, 1:5, 3:3	4:2	0:6	2:4, 1:5, 3:3	4:2	0:6
DAZ1/3 duplication	3:3	2:4, 1:5, 3:3	3:3:0	3:1:2	2:4, 1:5, 3:3	3:3	0:6	2:4, 1:5, 3:3	3:3	0:6
DAZ2/4 duplication	3:3	1:5, 0:6, 2:4	3:3:0	3:2:1	1:5, 0:6, 2:4	3:3	0:6	1:5, 0:6, 2:4	3:3	0:6
DAZ1/4 duplication	3:3	2:4, 1:5, 3:3	3:3:0	3:2:1	1:5, 0:6, 2:4	3:3	0:6	1:5, 0:6, 2:4	3:3	0:6
DAZ2/3 duplication	3:3	1:5, 0:6, 2:4	3:3:0	3:1:2	2:4, 1:5, 3:3	3:3	0:6	2:4, 1:5, 3:3	3:3	0:6

Each variant ratio indicated by the table at a given position is in accordance with the corresponding rearrangement subtype.
Variant ratios other than those indicated by the table are discordant with and contradict to the corresponding rearrangement subtype.
Variant ratios that are not only in accordance with but also indicate the assigned deletion or duplication subtype by themselves are written in red.

For the calculation of the above ratios, one deletion/duplication and one gene conversion was permitted. The conversion can take place before (starting variant ratio 2:2 or 0:4) or after (starting variant ratio 1:3) the large rearrangement. In the latter case, ratios written by smaller digits are expected as the result of the post-rearrangement gene conversion. Based on our observations, only DAZ3>DAZ4 conversion is allowed for the duplication of the class III DAZ3-specific markers (F-II 1005 and F-II 1053). As it can be seen, the applicability of class III variants is very limited since almost all variant ratios are in accordance with each rearrangement subtype.

[1]G_{2481} is supposed to be a class II/a DAZ1-specific marker according to the results of the cloning experiments (Supporting Table S6c-d).
[2]T_{111} is considered DAZ3-specific on the basis of the cloning experiments (Supporting Table S6c). When it is present, C_{111} is restricted to DAZ4.
[3]G_{1005} is a class III DAZ3-specific marker.
[4]C_{1053} is a class III DAZ3-specific marker.
