# Supplementary material for: Discrimination of Deletion and Duplication Subtypes of the Deleted in Azoospermia Gene Family in the Context of Frequent Interloci Gene Conversion
Source: PLoS One. 2016 Oct 10;11(10):e0163936. doi: 10.1371/journal.pone.0163936 (PMC5056753; doi:10.1371/journal.pone.0163936)
Supplement: S11 Table — (PDF) [file pone.0163936.s021.pdf]

Supporting Table S11a. Comparison of the variant ratios found in deletion samples Ydel_09 and Ydel_10 with those expected after a supposed DAZ2/DAZ4 deletion in control samples bearing different variant ratio haplotypes

		SFV position on Fragment I						SFV position on Fragment II										
Location		972	1209	1702	1820	1926	2481[2]	111[3]	978	1005	1053	1636	1646	1952	1961	1964[4]	1964[4]	2071
Marker specificity		DAZ1	DAZ2	DAZ2	DAZ4	DAZ1	-	-	DAZ4	DAZ3	DAZ3	DAZ2	DAZ3	DAZ3	DAZ3	DAZ3	DAZ4	DAZ4
Variants[1]		A:G	T:C	T:C	C:A	G:A	G:T	G:C(:T)	C:T	G:A	C:T	G:T	A:G	T:A	T:C	C:(A+G)	A:(C+G)	G:C
VRH	RefSeq	1:1	0:2	0:2	0:2	1:1	1:1	1:1	0:2	1:1	1:1	0:2	1:1	1:1	1:1	1:(0+1)	0:(1+1)	0:2
	3b	0:2	0:2	0:2	0:2	1:1	1:1[5]	1:1	0:2	1:1	1:1	0:2	1:1	1:1	1:1	1:(0+1)	0:(1+1)	0:2
	3a/1	0:2	0:2	0:2	0:2	1:1	1:1[5]	1:1	0:2	1:1	1:1	0:2	1:1	1:1	1:1	1:(0+1)	0:(1+1)	0:2
	3a/2	0:2	0:2	0:2	0:2	1:1	1:1[5]	1:0(:1)[7]	0:2	1:1	1:1	1:1[6]	1:1	1:1	1:1	1:(0+1)	0:(1+1)	0:2
	3a/3	0:2	0:2	0:2	0:2	1:1	1:1	1:1	0:2	1:1	1:1	0:2	1:1	1:1	1:1	1:(0+1)	0:(1+1)	0:2
	2	1:1	0:2	0:2	0:2	1:1	1:1	1:1	0:2	1:1	1:1	0:2	1:1	1:1	1:1	1:(0+1)	0:(1+1)	0:2
	1	1:1	0:2	0:2	0:2	1:1	1:1[5]	1:1	0:2	1:1	1:1	0:2	1:1	1:1	1:1	1:(0+1)	0:(1+1)	0:2
	4	0:2	0:2	0:2	0:2	1:1	1:1	N/A	0:2	0:2	0:2	0:2	1:1	1:1	1:1	1:(0+1)	0:(1+1)	0:2
	3c	0:2	0:2	0:2	0:2	1:1	1:1	N/A	0:2	0:2	0:2	0:2	1:1	1:1	1:1	1:(0+1)	0:(1+1)	0:2
Sample	Ydel_09	1:1	0:2	0:2	0:2	1:1	1:1	1:1	0:2	1:1	1:1	0:2	1:1	1:1	1:1	1:(0+1)	0:(1+1)	0:2
	Ydel_10	1:1	0:2	0:2	0:2	1:1	1:1	1:1	0:2	1:1	1:1	0:2	1:1	1:1	1:1	1:(0+1)	0:(1+1)	0:2

Conclusion: DAZ2/DAZ4 deletion of samples with VRH 1 and VRH 2 results in exactly the same series of variant ratios that found in samples Ydel_09 and Ydel_10. It supports the DAZ2/4 deletion status of these samples.

[1]Variants are arranged as family member-specific variant:non-specific variant at each SFV position.
[2]At position 2481 in Fragment I, there is no specific variant (DAZ1/2: G, DAZ3/4: T according to the human reference assembly).
[3]At position 111 in Fragment II, there is no specific variant (DAZ1/2: G, DAZ3/4: C according to the human reference assembly). In three samples (VRH 3a/2), T was found to replace one of the Cs. According to the results of the cloning experiments, T was located in DAZ3 (Supporting Table S6c).
[4]At position 1964, there is a DAZ3-specific C and a DAZ4-specific A according to the human reference assembly.
[5]Valid if G_{2481} is a class II/a DAZ1-specific marker, which is supported by the results of cloning and sequencing the four amplicons constituting Fragment I in samples 5466 and 6100 (Supporting Table S6c-d).
[6]Valid if the DAZ2-specific G_{1636} in Fragment II is transferred to DAZ1 in samples with VRH 3a/2, which is supported by the results of cloning and sequencing the four amplicons constituting Fragment II in sample 5466 (Supporting Table S6c).
[7]One G and one T are expected in VRH 3a/2, in contrast to one G and one C expected in the case of all other VRHs.

Supporting Table S11b. Comparison of the variant ratios found in deletion samples Ydel_09 and Ydel_10 with those expected after a supposed DAZ2/DAZ3 deletion in control samples bearing different variant ratio haplotypes

		SFV position on Fragment I						SFV position on Fragment II										
Location		972	1209	1702	1820	1926	2481[2]	111[3]	978	1005	1053	1636	1646	1952	1961	1964[4]	1964[4]	2071
Marker specificity		DAZ1	DAZ2	DAZ2	DAZ4	DAZ1	-	-	DAZ4	DAZ3	DAZ3	DAZ2	DAZ3	DAZ3	DAZ3	DAZ3	DAZ4	DAZ4
Variants[1]		A:G	T:C	T:C	C:A	G:A	G:T	G:C(:T)	C:T	G:A	C:T	G:T	A:G	T:A	T:C	C:(A+G)	A:(C+G)	G:C
VRH	RefSeq	1:1	0:2	0:2	1:1	1:1	1:1	1:1	1:1	0:2	0:2	0:2	0:2	0:2	0:2	0:(1+1)	1:(0+1)	1:1
	3b	0:2	0:2	0:2	0:2	1:1	1:1[5]	1:1	1:1	0:2	0:2	0:2	0:2	0:2	0:2	0:(0+2)	0:(0+2)	1:1
	3a/1	0:2	0:2	0:2	0:2	1:1	1:1[5]	1:1	1:1	0:2	0:2	0:2	0:2	0:2	0:2	0:(1+1)	1:(0+1)	1:1
	3a/2	0:2	0:2	0:2	0:2	1:1	1:1[5]	1:1[7]	1:1	0:2	0:2	1:1[6]	0:2	0:2	0:2	0:(1+1)	1:(0+1)	1:1
	3a/3	0:2	0:2	0:2	0:2	1:1	1:1	1:1	1:1	0:2	0:2	0:2	0:2	0:2	0:2	0:(1+1)	1:(0+1)	1:1
	2	1:1	0:2	0:2	1:1	1:1	1:1	1:1	1:1	0:2	0:2	0:2	0:2	0:2	0:2	0:(0+2)	0:(0+2)	1:1
	1	1:1	0:2	0:2	1:1	1:1	1:1[5]	1:1	0:2	1:1	1:1	0:2	1:1	1:1	1:1	1:(0+1)	0:(1+1)	0:2
	4	0:2	0:2	0:2	0:2	1:1	1:1	N/A	0:2	0:2	0:2	0:2	1:1	1:1	1:1	1:(0+1)	0:(1+1)	0:2
	3c	0:2	0:2	0:2	0:2	1:1	1:1	N/A	1:1	0:2	0:2	0:2	0:2	0:2	0:2	0:(0+2)	0:(0+2)	1:1
Sample	Ydel_09	1:1	0:2	0:2	0:2	1:1	1:1	1:1	0:2	1:1	1:1	0:2	1:1	1:1	1:1	1:(0+1)	0:(1+1)	0:2
	Ydel_10	1:1	0:2	0:2	0:2	1:1	1:1	1:1	0:2	1:1	1:1	0:2	1:1	1:1	1:1	1:(0+1)	0:(1+1)	0:2

Conclusion: There is no VRH among the controls whose DAZ2/DAZ3 deletion would result in the same series of variant ratios as that found in samples Ydel_09 and Ydel_10. It does not support the DAZ2/3 deletion status of these samples.

On the whole, the above two sets of comparison increase the probability of the DAZ2/4 deletion status of samples Ydel_09 and Ydel_10.

[1]Variants are arranged as family member-specific variant:non-specific variant at each SFV position.
[2]At position 2481 in Fragment I, there is no specific variant (DAZ1/2: G, DAZ3/4: T according to the human reference assembly).
[3]At position 111 in Fragment II, there is no specific variant (DAZ1/2: G, DAZ3/4: C according to the human reference assembly). In three samples (VRH 3a/2), T was found to replace one of the Cs. According to the results of the cloning experiments, T was located in DAZ3 (Supporting Table S6c).
[4]At position 1964, there is a DAZ3-specific C and a DAZ4-specific A according to the human reference assembly.
[5]Valid if G_{2481} is a class IIa DAZ1-specific marker, which is supported by the results of cloning and sequencing the four amplicons constituting Fragment I in samples 5466 and 6100 (Supporting Table S6c-d).
[6]Valid if the DAZ2-specific G_{1636} in Fragment II is transferred to DAZ1 in samples with VRH 3a/2, which is supported by the results of cloning and sequencing the four amplicons constituting Fragment II in sample 5466 (Supporting Table S6c).
